# Circular RNA circBNC2 inhibits epithelial cell G2-M arrest to prevent fibrotic maladaptive repair

**DOI:** 10.1038/s41467-022-34287-5

**Published:** 2022-10-31

**Authors:** Peng Wang, Zhitao Huang, Yili Peng, Hongwei Li, Tong Lin, Yingyu Zhao, Zheng Hu, Zhanmei Zhou, Weijie Zhou, Youhua Liu, Fan Fan Hou

**Affiliations:** 1 Division of Nephrology, Nanfang Hospital, Southern Medical University, State Key Laboratory of Organ Failure Research, National Clinical Research Center of Kidney Disease, Guangdong Provincial Key Laboratory of Renal Failure Research, Guangzhou, 510515 China; 2 Guangzhou Regenerative Medicine and Health Guangdong Laboratory, Guangzhou, 510515 China

**Keywords:** Acute kidney injury, Renal fibrosis, Mechanisms of disease, Molecular medicine

## Abstract

The mechanisms underlying fibrogenic responses after injury are not well understood. Epithelial cell cycle arrest in G2/M after injury is a key checkpoint for determining wound-healing leading to either normal cell proliferation or fibrosis. Here, we identify a kidney- and liver-enriched circular RNA, circBNC2, which is abundantly expressed in normal renal tubular cells and hepatocytes but significantly downregulated after acute ischemic or toxic insult. Loss of circBNC2 is at least partially mediated by upregulation of DHX9. Gain- and loss-of-function studies, both in vitro and in vivo, demonstrate that circBNC2 acts as a negative regulator of cell G2/M arrest by encoding a protein that promotes formation of CDK1/cyclin B1 complexes. Restoring circBNC2 in experimentally-induced male mouse models of fibrotic kidney and liver, decreases G2/M arrested cell numbers with secretion of fibrotic factors, thereby mitigating extracellular matrix deposition and fibrosis. Decreased expression of circBNC2 and increased G2/M arrest of epithelial cells are recapitulated in human ischemic reperfusion injury (IRI)-induced chronic kidney disease and inflammation-induced liver fibrosis, highlighting the clinical relevance. These findings suggest that restoring circBNC2 might represent a potential strategy for therapeutic intervention in epithelial organ fibrosis.

## Abstract (web summary)

G2/M arrest of epithelial cells leads to fibrosis with unclear mechanisms. This study identifies a protein-encoding circRNA, circBNC2, which inhibits epithelial cells G2/M arrest to prevent fibrotic maladaptive repair in damaged kidney and liver, revealing a potential intervention target for fibrosis.

## Introduction

Tissue fibrosis can affect many organs and is responsible for up to 45% of all deaths in the developed world^1^. Fibrosis, characterized by the excessive accumulation of extracellular matrix (ECM), often occurs as a wound-healing response to chronic tissue inflammation or injury, which can lead to permanent scarring, organ dysfunction and, ultimately, death, as seen in end-stage renal disease and liver cirrhosis^2,3^. Although ECM accumulation has been considered to be a typical response to wounding, normal tissue repair can evolve into a progressively fibrotic response if the injury is severe or repetitive, or if the wound-healing response itself becomes dysregulated^4^.

Epithelial cells, such as renal tubule epithelial cells (TECs) and hepatocytes, comprise the bulk of the organ parenchyma. These cells are the primary targets of a variety of ischemic or toxic insults^5^, and are largely responsible for repairing the tissues when they survive an acute injury^6^. Non-fibrotic repair depends on efficient de-differentiation, proliferation and re-differentiation of the surviving epithelial cells^7^. However, increasing evidence has demonstrated that, in many cases, the repair process is incomplete; severe injury leads to maladaptive repair with persistent parenchymal inflammation and excessive ECM deposition^6^. One example is that severe acute kidney injury can result in renal fibrosis through triggering cell cycle arrest of proximal TECs in the G2/M phase with secretion of profibrotic factors^7–9^. Similarly, oxidative stress promotes hepatocyte apoptosis through inducing cell cycle arrest^10,11^. These data suggest that maladaptive epithelial cell repair is implicated in organ fibrosis. However, the exact cellular and molecular mechanisms involved in regulating fibrotic maladaptive repair are not well understood.

Emerging evidence has correlated circular RNAs (circRNAs) with fibrogenesis in the kidney and liver^12,13^. CircRNAs, circular molecules covalently closed at their 5’ and 3’ ends, are more resistant to exonucleases than linear RNAs^14,15^. CircRNAs act through sponging microRNAs, regulate RNA transcription and mRNA splicing, and modulate protein translation and degradation into peptides^16–18^. Although the tissue-specific expression of circRNAs has been linked to organ fibrosis, the role of circRNAs in regulating fibrotic maladaptive repair remains largely unknown.

In this study, we identified a kidney- and liver-enriched circRNA, circBNC2, which acted as a negative regulator of the G2/M cell cycle arrest via encoding a protein that mediated formation of CDK1/cyclin B1 complexes, a specific promoter for cell entry into mitosis^19,20^. circBNC2 conferred protective effects in terms of mitigating maladaptive repair and consequent fibrosis after severe kidney and liver injury. This discovery adds to our understanding of the complex wound-healing response and suggests that restoring circBNC2 might be a potential strategy for therapeutic intervention in epithelial organ fibrosis.

## Results

### circBNC2 is abundantly expressed in normal kidney and liver and downregulated after severe injury

To study the role of circRNAs in renal TECs after injury, we established two renal ischemia-reperfusion injury (IRI) models in mice. Ischemia was induced by either clipping bilateral renal pedicles for 20 min (mild IRI) or for 40 min (severe IRI). As previously reported^7,21,22^, persistent renal dysfunction with renal fibrosis was observed in severe but not mild IRI animal models. (Supplementary Fig. 1a–d).

**Fig. 1:**
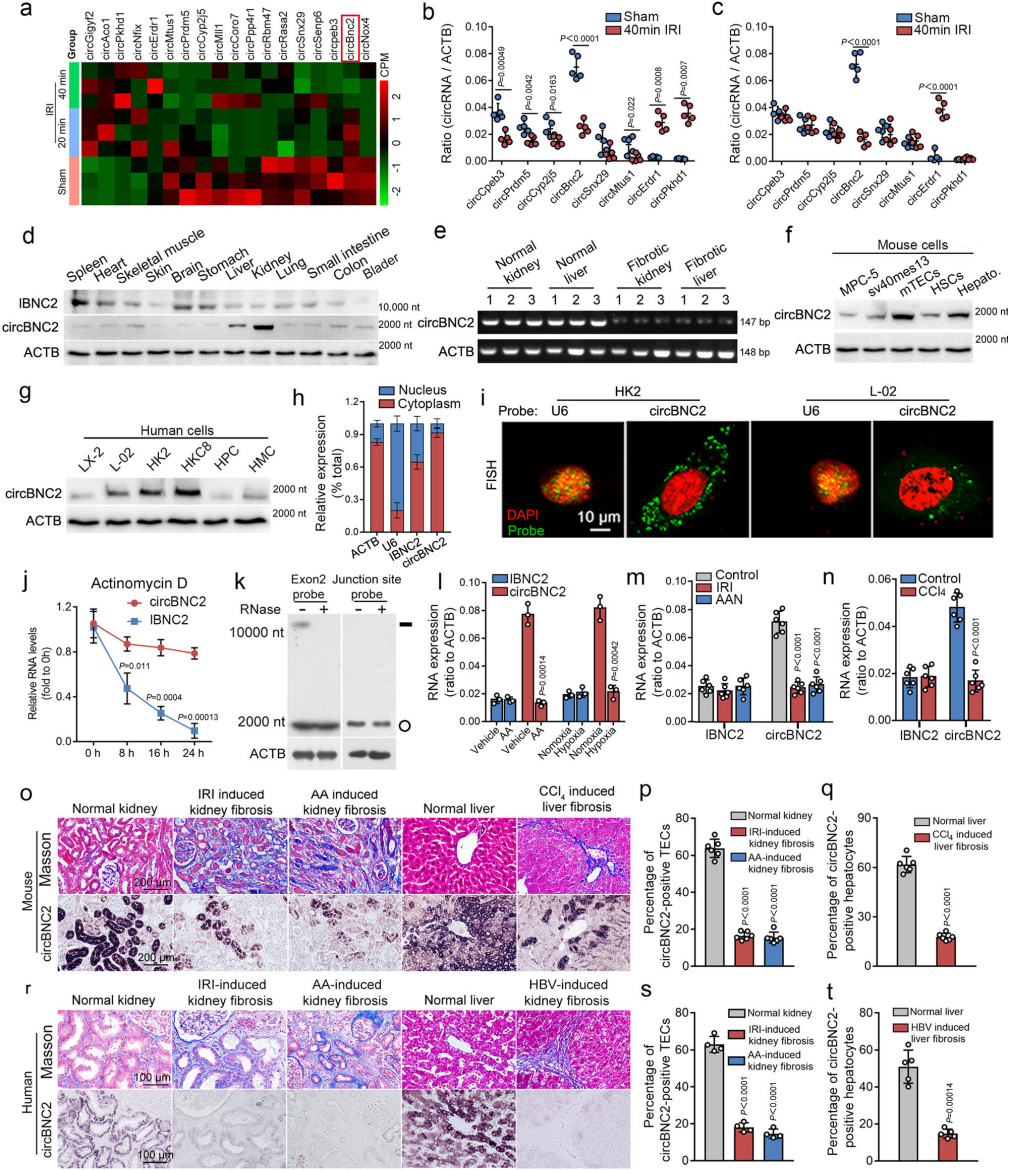
circBNC2 is abundantly expressed in normal kidney and liver and downregulated after severe injury. **a** Heatmap showing changes in expression levels of circRNAs >1.5-fold of Sham in TECs isolated from mice treated with mild- (20-min ischemia) or severe IRI (40-min ischemia) (*n* = 3 mice in each group). See also Supplementary Fig. 1e–g. **b**, **c** Expression of 8 circRNAs differentially expressed in RNA-sequencing between severe IRI and Sham mice, determined by quantitative RT-PCR (qRT-PCR) at day 1 (**b**) or day 14 (**c**) after IRI (*n* = 5 mice in each group). **d** The RNA levels of circBNC2 and linearized BNC2 (lBNC2), examined by northern blots in various mouse tissues. **e** RT-PCR showing abundant circBNC2 in homogenates of normal kidney and liver, and downregulated expression of circBNC2 in human IRI-induced fibrotic kidney and in hepatitis B virus-induced liver fibrosis (*n* = 3 in each group). **f** The expression of circBNC2, detected by northern blots, was abundant in mouse TECs and primary hepatocytes, and weak in mesangial cells (sv40mes13), podocytes (MPC-5) and primary stellate cells (HSCs). **g** The expression of circBNC2, detected by northern blots, was mainly observed in human TECs (HKC8 and HK2) and hepatocytes (L-02), and weak in mesangial cells (HMC), podocytes (HPC) and stellate cells (LX-2). **h** qRT-PCR showed that circBNC2 and lBNC2 expressed in the cytoplasmic of normal TECs (HK2). ACTB or U6 was the reference gene in cytoplasmic or nuclear part of TECs, separately. **i** Confocal FISH images showing cytoplasmic localization of circBNC2 in human TECs (HK2) and hepatocytes (L-02) cells. **j** The relative RNA levels of circBNC2 and lBNC2 assayed by qRT-PCR in HK2 treated with actinomycin D. **k** Northern blots using the Exon 2 probe (detecting circBNC2 and lBNC2) or junction-specific probe (detecting circBNC2) in total RNAs, in the presence or absence of RNase treatment. Total RNAs were extracted from HK2 cells. **l** qRT-PCR showing circBNC2 and lBNC2 expression in HK2 cells exposed to hypoxia or Aristolochic acids (AA) for 24 h. **m**, **n** qRT-PCR showing circBNC2 and lBNC2 expression in IRI- or AA-induced kidney fibrosis and CCl_4_-induced liver fibrosis in mice (*n* = 6 in each group). **o**–**q** In situ hybridization showing circBNC2 expression in IRI- or AA-induced kidney fibrosis and CCl_4_-induced liver fibrosis (**o**) and the quantification data (**p**, **q**) (*n* = 6 in each group). **r**–**t** In situ hybridization showing circBNC2 expression in IRI- or AA-induced human kidney fibrosis (*n* = 4 in each group) and HBV-induced liver fibrosis (*n* = 5 in each group) (**r**) and the quantification data (**s**, **t**). For **h**, **j**, **l**, *n* = 3 biologically independent cells. Data are expressed as means ± SD. Two-sided *T*-test was used for the comparison of two groups (**b**, **c**, **j**, **l**, **n**, **q**, **t**). One-way ANOVA with Bonferroni post hoc test was used for comparison among multiple groups (**m**, **p**, **s**). Source data are provided as a Source Data file.

To screen for differentially expressed circRNAs between IRI and sham-treated (Sham) mice, we performed high-throughput sequencing with tubules isolated from IRI and Sham mice. A total of 8 dysregulated circRNAs were identified in severe IRI mice, of which 2 circRNAs were upregulated and 6 circRNAs were downregulated, as compared to those in the Sham mice (Fig. 1a and Supplementary Fig. 1e–g). circBNC2 was derived from exon 2 of the *BNC2* gene, which was verified by Sanger DNA sequencing. The downregulation of circBNC2 persisted for >14 days after severe IRI (Fig. 1b, c). However, dysregulation of circBNC2 was not identified in mice with mild IRI injury (Supplementary Fig. 1i–k).

To characterize circBNC2, we examined the expression profiles of circBNC2 and its linearized form (lBNC2) separately across normal tissues from mice. The results showed that circBNC2, but not lBNC2, was abundantly expressed in the kidneys and, to a lesser extent, in the liver (Fig. 1d). Elevated expression of circBNC2 was also observed in normal human kidneys and liver (Fig. 1e). Northern blots of various cell lines derived from mice and humans demonstrated that circBNC2 was expressed mainly in renal TECs and hepatocytes (Fig. 1f, g), and was cytoplasmically localized (Fig. 1h, i). As observed for other circRNAs, circBNC2 was more stable than lBNC2 and resistant to actinomycin D and RNAse treatment (Fig. 1j, k). The sequences of circBNC2 had 91% homology between human and mouse (Supplementary Fig. 1l). When TECs were exposed to hypoxia or aristolochic acid (AA) for 24 h, expression of circBNC2 was dramatically decreased (Fig. 1l and Supplementary Fig. 1m). A similar phenomenon was observed in hypoxia-treated hepatocytes (Supplementary Fig. 1n).

To further confirm the dysregulation of circBNC2 in vivo, the expression of circBNC2 was examined in mouse models with IRI- and aristolochic acids (AA)-induced kidney fibrosis and carbon tetrachloride (CCl_4_)-induced liver fibrosis. Compared to that in normal kidney and liver tissues, expression of circBNC2 was remarkably downregulated in the mouse fibrotic kidney and liver (Fig. 1m–q). These findings were recapitulated in human IRI- or AA-induced chronic kidney disease and inflammation-induced liver fibrosis (Fig. 1e, r–t).

### Upregulation of DHX9 causes downregulation of circBNC2

**Fig. 2:**
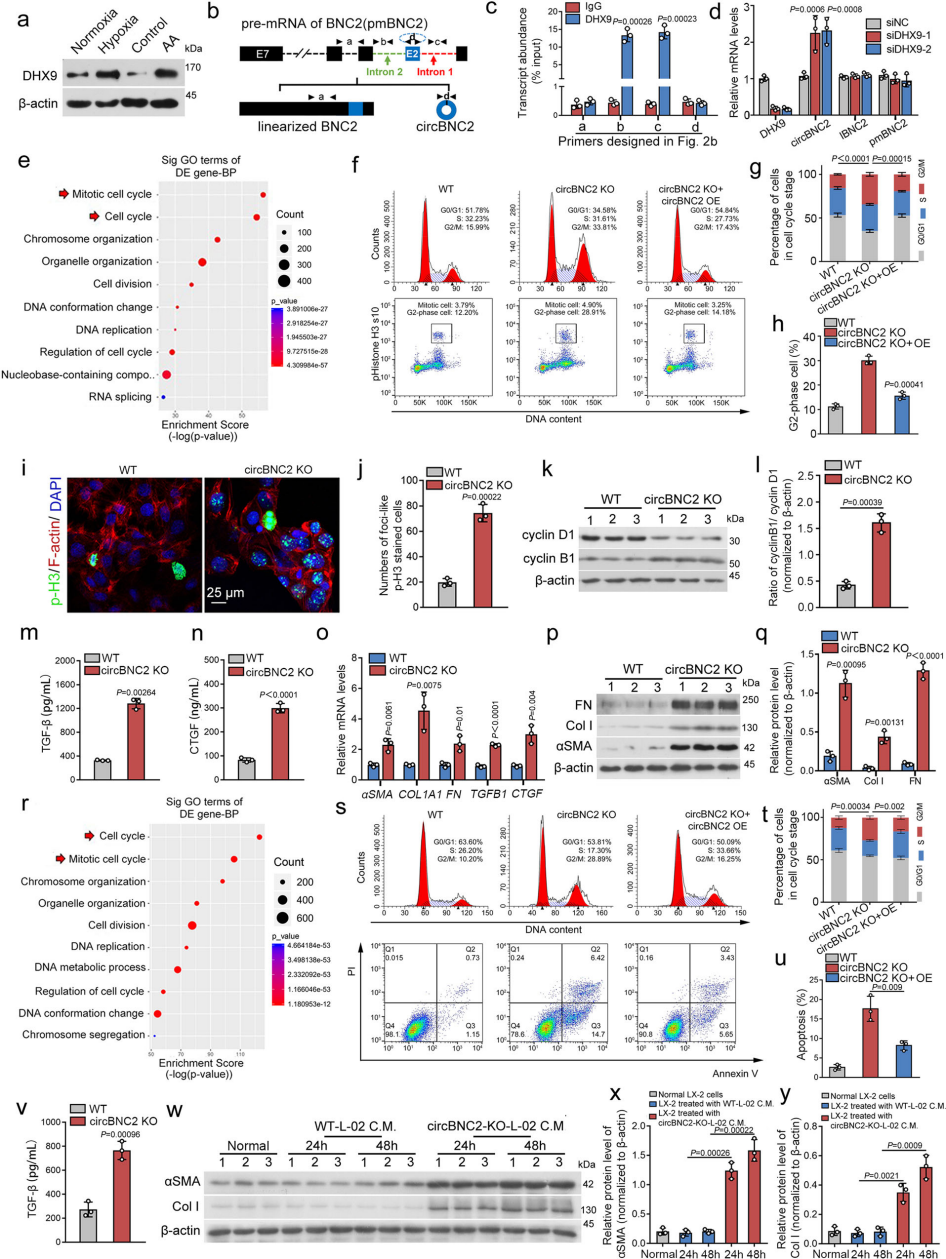
Downregulation of circBNC2 promotes epithelial cell G2/M arrest after injury. **a** Western blots showing DHX9 expression in lysates from HK2 cells exposed to hypoxia or AA for 24 h. **b, c** The primer sets designed in the pre-mRNA of BNC2 precursor (**b**) and the transcript abundance of amplicons a-d relative to input, detected by RNA immunoprecipitation with anti-DHX9 in lysates from HK2 cells treated with hypoxia for 24 h, followed by qRT-PCR assay (**c**). **d** qRT-PCR showing DHX9, circBNC2, lBNC2 and pre-mRNA of BNC2 (pmBNC2) expression in 24-h hypoxia-treated HK2 cells transfected with siRNAs targeting DHX9. **e** The top 10 significantly enriched Gene Ontology (GO) terms of the differentially expressed genes in mRNA sequencing of circBNC2-KO HK2 cells compared to wild-type (WT). **f**–**h** Cell cycle analysis by flow cytometry in HK2 cells, showing knockout of circBNC2 induced G2/M cell cycle arrest (**f**, **g**), especially G2 phase cell (**h**). **i**, **j** Immunofluorescence staining for p-H3 in circBNC2-KO HK2 cells showing increase in G2 phase positive cells (**i**) and the quantification data (**j**). See also Supplementary Fig. 2n. **k**, **l** Western blots showing cyclin B1 and cyclin D1 expression in circBNC2-KO HK2 cells (**k**) and the ratio of cyclin B1/cyclin D1 (**l**). **m**, **n** Secretion of TGF-β1 (**m**) and CTGF (**n**) by circBNC2-KO HK2 cells was examined by ELISA. **o** qRT-PCR showing mRNA levels of *αSMA*, *COL1A1*, *FN*, *TGFB1* and *CTGF* expression in circBNC2-KO HK2 cells. **p**, **q** Western blots showing protein levels of αSMA, Col I, and FN in circBNC2-KO HK2 cells (**p**), and the quantification data (**q**). **r** The top 10 significantly enriched Gene Ontology (GO) terms of the differentially expressed genes in mRNA sequencing of circBNC2-KO L-02 cells compared to wild-type. **s**–**u** Cell cycle analysis by flow cytometry in L-02 cells showing knockout of circBNC2 induced G2/M cell cycle arrest (**s, t**), especially G2 phase cell cycle arrest (**u**). While overexpression of circBNC2 partially rescued the G2/M cell cycle arrest in circBNC2-KO cells. **v** Levels of TGF-β1 in supernatants of circBNC2-KO L-02 cells, examined by ELISA. **w**–**y** Western blots showing protein levels of αSMA and Col I in LX-2 cells incubated with conditional medium (C.M.) from circBNC2-KO L-02 cells or WT L-02 cells for 24 or 48 h. For **c**, **d**, **g**, **h**, **j**, **l**–**o**, **q**, **t**–**v**, **x**, **y**, *n* = 3 biologically independent cells. Data are expressed as means ± SD. Two-sided *T*-test was used for the comparison of two groups (**c**, **h**, **j**, **l**, **m**, **n**, **o**, **q**, **u**, **v**, **x**, **y**). One-way ANOVA with Bonferroni post hoc test was used for comparison among multiple groups (**d**, **g**, **t**). Source data are provided as a Source Data file.

To explore the mechanisms underlying circBNC2 downregulation, we examined the expression of DExH-Box Helicase 9 (DHX9) in lysates of TECs subjected to hypoxia or AA for 24 h, and in lysates of kidney tissue from mice subjected to IRI- or AA-treatment. DHX9 is a nuclear RNA helicase that inhibits the formation of circRNAs by binding to their flanking inverted complementary sequences^23^. We found DHX9 was significantly upregulated in vitro and in vivo after injury (Fig. 2a and Supplementary Fig. 2a–c). By analyzing cross-linking immunoprecipitation-high-throughput sequencing (CLIP-sequencing) data^23^, we found that DHX9 could bind to the flanking introns of the unspliced BNC2 precursor (Supplementary Fig. 2d). RNA immunoprecipitation assays involving DHX9 confirmed that DHX9 bound to introns 1 and 2 of the circBNC2 precursor (Fig. 2b, c). Furthermore, knocking-down DHX9 with siRNAs upregulated circBNC2 expression in TECs subjected to 24-h hypoxia-treated TECs, but did not affect the expression of lBNC2 and pre-mRNA of BNC2 (Fig. 2d). A Previous study reports that DHX9 functions together with ADAR1 when suppressing *Alu*-mediated circRNA biogenesis^23^. To explore the expression and function of ADAR1 in relation to DHX9 in this study, we first examined the expression of ADAR1 in damaged TECs in vitro and in vivo. The results showed that ADAR1 expression remained unchanged in injured TECs (Supplementary Fig. 2e–h). We next investigated whether ADAR1 regulated circBNC2 expression by knocking-down ADAR1 with siRNAs in 24-h hypoxia-treated TECs. The results showed that knocking-down ADAR1 promoted circBNC2 expression (Supplementary Fig. 2i). To uncover the potential mechanisms of ADAR1-induced circBNC2 downregulation in injured TECs, we found that DHX9 interacted with the p150 isoform of ADAR1 (Supplementary Fig. 2j). Therefore, we supposed that ADAR1 might guide DHX9 to the intronic regions of circBNC2 precursor. RNA immunoprecipitation assays were performed in ADAR1-depleted TECs subjected to 24-h hypoxia, and the results demonstrated that knockdown of ADAR1 inhibited the binding of DHX9 to introns 1 and 2 of the circBNC2 precursor (Supplementary Fig. 2k).

### Downregulation of circBNC2 promotes epithelial cell G2/M arrest after injury

To study the biological functions of circBNC2 in epithelial cells, we applied CRISPR-cas9 editing to the intronic region of BNC2 pre-mRNA to knock-out circBNC2, while preserving the expression of BNC2 mRNA and protein (Supplementary Fig. 2l, m). The biological processes that circBNC2 may be involved in were analyzed using mRNA sequencing and gene ontology analysis in circBNC2-knockout (KO) cells. The results showed that the most significant function of circBNC2 in TECs was mitotic cell cycle regulation (Fig. 2e). Compared to those in wild-type (WT) TECs, circBNC2-KO cells presented an increase in G2/M-arrested cells, particularly G2 phase cells by flow cytometry analysis and immunofluorescence of p-histone H3 (Fig. 2f–j and Supplementary Fig. 2n), and an elevated ratio of cyclin B1/cyclin D1 (Fig. 2k, l), a marker of G2/M cell cycle arrest. Furthermore, restoring expression of circBNC2 in circBNC2-KO TECs (Supplementary Fig. 2o) clearly decreased the percentage of G2/M arrested cells (Fig. 2f–h). Loss-of-function in circBNC2 increased the secretion of profibrotic factors, including transforming growth factor β1 and connective tissue growth factor, thereby increasing ECM production at both the mRNA and protein levels (Fig. 2m–q).

To determine whether the BNC2 protein regulates G2/M cell cycle arrest, we depleted BNC2 protein but preserved the expression of circBNC2 by using a CRISPR-cas9-induced frameshift mutation in exon 1 of the BNC2 pre-mRNA (Supplementary Fig. 2p, q). As shown in Supplementary Fig. 2, depleting BNC2 protein had no effect on the cell cycle (Supplementary Fig. 2r–v).

Gene ontology analysis in circBNC2-KO human hepatocytes presented results similar to those of circBNC2-KO TECs (Fig. 2r). Loss-of-function in circBNC2 increased G2/M cell cycle arrest in hepatocytes, with increased cell apoptosis (Fig. 2s–u). Restoring expression of circBNC2 in circBNC2-KO hepatocytes (Supplementary Fig. 2w) rescued cell cycle arrest and mitigated cell apoptosis (Fig. 2s–u). Compared to supernatants from WT hepatocytes, supernatants from circBNC2-KO hepatocytes contained higher levels of TGF-β1 (Fig. 2v). When hepatic stellate cells were cultured in vitro with such conditioned medium, the levels of α-smooth muscle actin (αSMA) and collagen I (Col I) in stellate cells were significantly increased compared to stellate cells cultured with conditioned medium from WT hepatocytes (Fig. 2w–y).

### Ectopic expression of circBNC2 improves epithelial cell G2/M arrest after injury

**Fig. 3:**
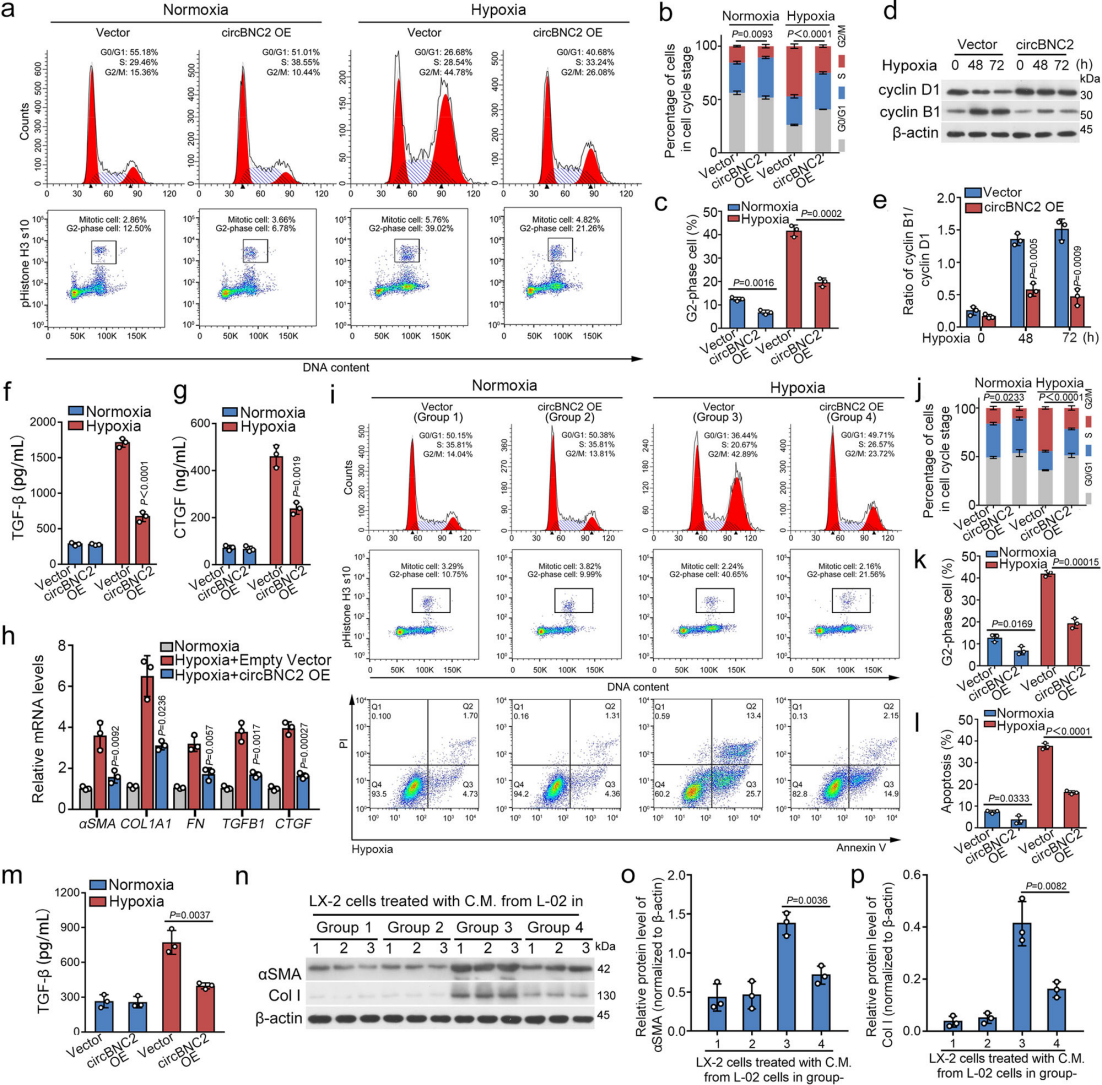
Ectopic expression of circBNC2 attenuates epithelial cell G2/M arrest after injury. **a**–**c** Cell cycle analysis by flow cytometry in HK2 cells showing overexpression of circBNC2 reduced 72-h hypoxia-induced G2/M cell cycle arrest (**a**, **b**), especially G2 phase cell cycle arrest (**c**). **d**, **e** Western blots showing the expression of cyclin B1 and cyclin D1 (**d**) and the quantification of cyclin B1/cyclin D1 (**e**). **f**, **g** Secretion of TGF-β1 (**f**) and CTGF (**g**), determined by ELISA, in 72 h hypoxia-treated HK2 cells transfected with circBNC2 or empty vector. **h** qRT-PCR showing mRNA levels of profibrotic factors expression in 72 h hypoxia-treated HK2 cells transfected with circBNC2 or empty vector. **i**–**l** Cell cycle analysis by flow cytometry in L-02 cells showing overexpression of circBNC2 inhibited G2/M cell cycle arrest and cell apoptosis induced by 72-h hypoxia treatment. The supernatants from L-02 cells, grouped as the same in this experiment, were collected to treat LX-2 cells, separately, the results were presented in panel **n**. **m** Levels of TGF-β1, examined by ELISA, in 72 h hypoxia-treated L-02 cells transfected with circBNC2 or empty vector. **n**–**p** Western blots showing protein levels of αSMA and Col I in LX-2 cells incubated with conditional medium from 72 h hypoxia-treated L-02 cells transfected with circBNC2 or empty vector. L-02 cells in Group 1–4 of this experiment were the same as the L-02 cells with different treatments in panel **i**. For **b**, **c**, **e**–**h**, **j**–**l**, **m**, **o**, **p**, *n* = 3 biologically independent cells. Data are expressed as means ± SD. Two-sided *T*-test was used for the comparison of two groups (**b**, **c**, **e**, **f**, **g**, **h**, **j**, **k**, **l**, **m**, **o**, **p**). Source data are provided as a Source Data file.

To further confirm the regulatory effects of circBNC2 on cell cycle arrest, we ectopically expressed circBNC2 in human TECs before injury (Supplementary Fig. 3a–d). Gain-of-function in circBNC2 inhibited G2/M arrest of human TECs induced by 72-h of hypoxia treatment (Fig. 3a–c) or AA exposure (Supplementary Fig. 3e–g), decreased the ratio of cyclin B1/cyclin D1 in damaged TECs (Fig. 3d, e and Supplementary Fig. 3h, i), and inhibited secretion of profibrotic factors in TECs after injury (Fig. 3f, g and Supplementary Fig. 3j, k), thus leading to decreased mRNA levels of profibrotic factors (Fig. 3h and Supplementary Fig. 3l).

Similarly, gain-of-function in circBNC2 improved G2/M cell cycle arrest, reduced apoptotic cell numbers, and decreased TGF-β1 secretion in 72-h hypoxia-treated hepatocytes (Fig. 3i–m). Activation of hepatic stellate cells was significantly inhibited when the cells were cultured with conditioned medium from circBNC2-overexpressing hepatocytes, as compared to hepatic stellate cells cultured with conditioned medium from hepatocytes expressing an empty vector only. (Fig. 3n–p).

### circBNC2 promotes CDK1/cyclin B1 complex formation via encoding a 681-amino-acid protein

**Fig. 4:**
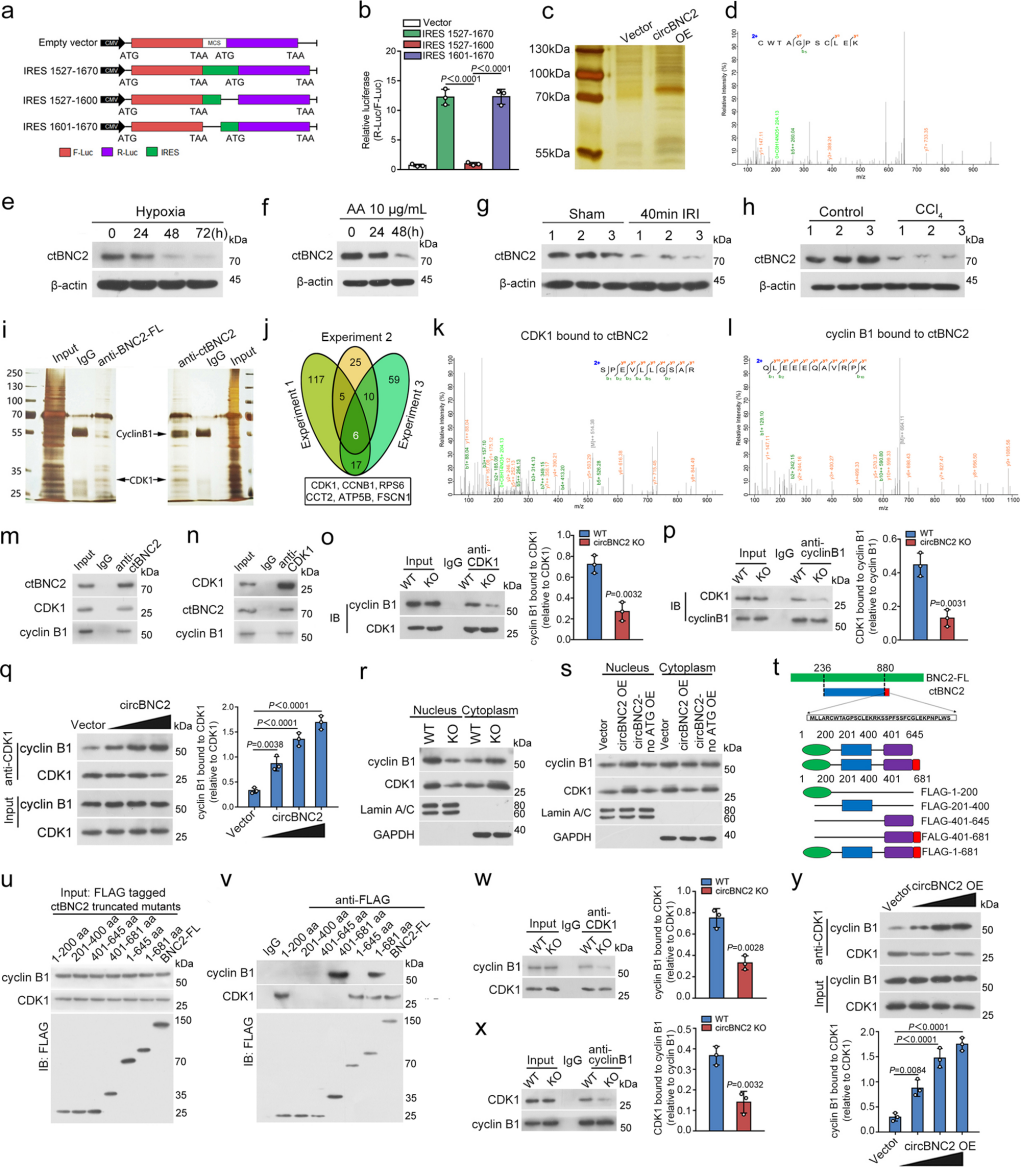
circBNC2 promotes CDK1/cyclin B1 complex formation via encoding a 681-amino acid protein. **a**, **b** IRES sequences in circBNC2 or its truncations were cloned between R-luc and F-Luc reporter genes (**a**) The predicted IRES activity in circBNC2 was tested by luciferase reporter assays (**b**). **c** Silver-staining of proteins from lysates of HK2 cells transfected with circBNC2 or empty vector. LC-MS analysis (samples from 3 independent experiments, *n* = 1 for each experiment) was used with the gel bands (70–100 kDa) from the lysates of HK2 cells overexpressing circBNC2. **d** Mass spectrometry result identifying a specific peptide sequence for circRNA translated BNC2 (ctBNC2). See also Supplementary Fig. 4a. **e**, **f** Western blots showing ctBNC2 expression in HK2 cells exposed to hypoxia (**e**) or AA (**f**) for the indicated timepoints. **g**, **h** Western blots showing ctBNC2 expression in cortex homogenates from mice with IRI-induced kidney fibrosis (**g**) and in liver homogenates from mice with CCl_4_-induced liver fibrosis (**h**). **i** The proteins in lysates of HK2 cells precipitated with anti-ctBNC2 or anti-full-length BNC2 (BNC2-FL) were detected by immunoprecipitation using normal IgG as the negative control. The SDS-PAGE gel with abundant bands (25–35 and 55–70 kDa) were collected and analyzed by Mass spectrometry to identify proteins interacted with ctBNC2 or BNC2-FL (3 independent experiments, *n* = 1 for each experiment). **j** A Venn diagram showing the intersection presenting 6 proteins (CDK1, cyclin B1, RPS6, ATP5B, CCT2 and Fascin) bound to ctBNC2 in three replicated experiments. **k**, **l** CDK1 and cyclin B1 bound to ctBNC2, as shown by Mass spectrometry results. See also Supplementary Data 1. **m**, **n** Interaction of ctBNC2 with CDK1 and cyclin B1, as shown by western blotting following immunoprecipitation of either ctBNC2 (**m**) or CDK1 (**n**). **o**, **p** Interaction of CDK1 with cyclin B1 was inhibited in circBNC2-KO HK2 cells, as shown by western blotting following immunoprecipitation of either CDK1 (**o**) or cyclin B1 (**p**). **q** Interaction of CDK1 with cyclin B1 was increased by overexpressing circBNC2 in 24-h hypoxia-treated HK2 cells in a dose-dependent manner, as shown by Immunoprecipitation assay followed by western blot. **r** Western blots showing CDK1 and cyclin B1 nuclear translocation was inhibited in circBNC2-KO HK2 cells. GAPDH, glyceraldehyde-3-phosphate dehydrogenase. **s** Western blots showing CDK1 and cyclin B1 nuclear translocation in circBNC2 or circBNC2^no ATG^ overexpressed HK2 cells treated by hypoxia for 24 h. **t** To test the binding site of ctBNC2 with CDK1 and cyclin B1, deletion mutants of ctBNC2 were established and tagged with FLAG. See also Supplementary Table 1. **u**, **v** Interaction of CDK1, cyclin B1 with ctBNC2 truncated mutants in vitro as shown by western blotting following immunoprecipitation of FLAG. **w**, **x** Interaction of CDK1 with cyclin B1 was inhibited in circBNC2-KO L-02 cells, as shown by western blotting following immunoprecipitation of either CDK1 (**w**) or cyclin B1 (**x**). **y** Interaction of CDK1 with cyclin B1 was increased by overexpressing circBNC2 in 24-h hypoxia-treated L-02 cells in a dose-dependent manner, as shown by western blots. For **b**, **o**, **p**, **q**, **w**–**y**, *n* = 3 biologically independent cells. Data are expressed as means ± SD. Two-sided *T*-test was used for the comparison of two groups (**o**, **p**, **w**, **x**). One-way ANOVA with Bonferroni post hoc test was used for comparison among multiple groups (**b**, **q**, **y**). Source data are provided as a Source Data file.

Based on the Coding Potential Calculator (CPC)^24^, circBNC2 has an open reading frame (ORF), predicted to encode a 681-amino acid (aa) protein (which we termed circBNC2-translated protein (ctBNC2)) (Supplementary Fig. 4a). This ORF is driven by an internal ribosome entry site (IRES) (Supplementary Fig. 4a), of which the activity was validated by luciferase reporter assays (Fig. 4a, b). Western blots with anti-FLAG antibody verified a 75 kDa protein in cells transfected with FLAG-tagged circBNC2 plasmids (Supplementary Fig. 4b). By performing mass spectrometry with lysates from circBNC2-overexpressing cells, we identified a specific peptide sequence for ctBNC2 (Fig. 4c, d). We next generated an anti-ctBNC2 antibody, which recognized a similar single band in circBNC2-transfected TECs. Western blotting using this antibody revealed increased expression of ctBNC2 in normal TECs overexpressing circBNC2, as compared to cells overexpressing the empty vector or circBNC2-ATG-deleted mutants (Supplementary Fig. 4c). Furthermore, overexpressing ATG-deleted circBNC2 did not rescue G2/M cell cycle arrest in human TECs exposed to hypoxia for 72 h (Supplementary Fig. 4d–f). These data suggested that circBNC2 encoded a previously undiscovered protein, which might act as a key functional regulator of the cell cycle.

Interestingly, downregulation of ctBNC2 was found in human TECs after hypoxia or AA exposure in vitro (Fig. 4e, f), and in mouse IRI-induced fibrotic kidneys, as well as in CCl_4_-induced liver fibrosis (Fig. 4g, h). To explore the function of ctBNC2, we performed immunoprecipitation-mass spectrometry (IP-MS) to identify the proteins that could interact with ctBNC2 or full-length BNC2 protein (BNC2-FL) using IgG as a negative control. SDS-PAGE revealed abundant bands (25–35 and 55–70 kDa), which were collected and analyzed by MS to identify proteins that interacted with ctBNC2 or BNC2-FL. This experiment was replicated three times. The lists of proteins that interacted with ctBNC2 or BNC2-FL, but not with IgG, are presented in Supplementary Data 1 and 2, separately. As shown in Fig. 4j–l and Supplementary Fig. 4g, six proteins (CDK1, cyclin B1, RPS6, ATP5B, CCT2 and Fascin) were found to interact with ctBNC2, and one protein CDK1 was found to interact with BNC2-FL in three replicate experiments, separately (Fig. 4j and Supplementary Fig. 4g).

To further confirm the specific role of interactions involving ctBNC2 and the binding proteins in regulating cell cycle in TECs, we knocked-down these six proteins bound to ctBNC2 or BNC2-FL with siRNAs, separately. Knocking-down cyclin B1 or CDK1 induced obvious G2/M cell cycle arrest, while knocking-down RPS6, ATP5B, CCT2 and Fascin resulted in only mild G0/G1 cell cycle arrest (Supplementary Fig. 4h, i). Furthermore, depleting BNC2-FL protein had no effect on cell cycle regulation (Supplementary Fig. 2r–v). These data suggest that the regulatory effect on G2/M cell cycle arrest is ctBNC2 specific and depends on both CDK1 and cyclin B1.

CDK1/cyclin B1 complex formation and nuclear translocation has been demonstrated to play a critical role in the initiation of mitosis^19,20^. To investigate whether ctBNC2 was involved in CDK1/cyclin B1 complex formation, co-immunoprecipitation assays were performed. The data demonstrated that ctBNC2 interacted with both CDK1 and cyclin B1 (Fig. 4m, n). Consistently, knocking-out circBNC2 in vitro decreased the interaction between CDK1 and cyclin B1 in normal human TECs (Fig. 4o, p), while overexpression of circBNC2 promoted such interactions between CDK1 and cyclin B1 in 24-h hypoxia-treated TECs (Fig. 4q). Consistently, knocking-out circBNC2 in normal TECs inhibited nuclear localization of CDK1/cyclin B1 complexes (Fig. 4r), while overexpression of circBNC2 promoted the nuclear translocation of the same in hypoxia-treated TECs (Fig. 4s).

To determine the ctBNC2 binding sites of CDK1 and cyclin B1, we generated full-length or truncated ctBNC2 constructs (Fig. 4t and Supplementary Table 1). Co-immunoprecipitation assays demonstrated that ctBNC2 bound to CDK1 with its 1–200 aa sequence and bound to cyclin B1 with its 401–681 aa sequence, separately, whereas BNC2-FL could bind to CDK1 but not cyclin B1 (Fig. 4u, v). Similarly, co-immunoprecipitation assays showed that knocking-out circBNC2 in normal hepatocytes decreased interactions between CDK1 and cyclin B1 (Fig. 4w, x), while overexpression of circBNC2 promoted the formation of CDK1/cyclin B1 complexes in hypoxia-treated hepatocytes (Fig. 4y).

To further investigate whether ctBNC2 or BNC2-FL was the critical element for interactions involving CDK1 and cyclin B1 to promote progression through G2 to M phase of the cell cycle in injured TECs, TECs were ectopically overexpressed with ctBNC2 or BNC2-FL, then subjected to 72-h of hypoxia. We found that ctBNC2 overexpression promoted the interaction between CDK1 and cyclin B1 in a dose-dependent manner (Supplementary Fig. 4j, k), while BNC2-FL overexpression had no effect on interactions between cyclin B1 and CDK1 (Supplementary Fig. 4l, m). Consistently, ctBNC2 overexpression alleviated G2/M cell cycle arrest induced by 72-h hypoxia treatment in TECs, whereas overexpressing BNC2-FL did not (Supplementary Fig. 4n–p).

### circBNC2 alleviates fibrotic maladaptive repair in kidney after injury

In severe IRI (40-min ischemia)-induced fibrotic kidney, downregulation of circBNC2 was observed 1-day post injury and persisted for >14 days. The number of phosphorylated-histone 3 (p-H3)-stained cells, a marker of G2/M-phase cells, increased at day 3 post injury, with an increase in profibrotic factors (Supplementary Fig. 5a–e). However, mild IRI (20-min ischemia) did not affect the number of p-H3-positive cells or the ratio of cyclin B1/cyclin D1 (Supplementary Fig. 5f–i).

**Fig. 5:**
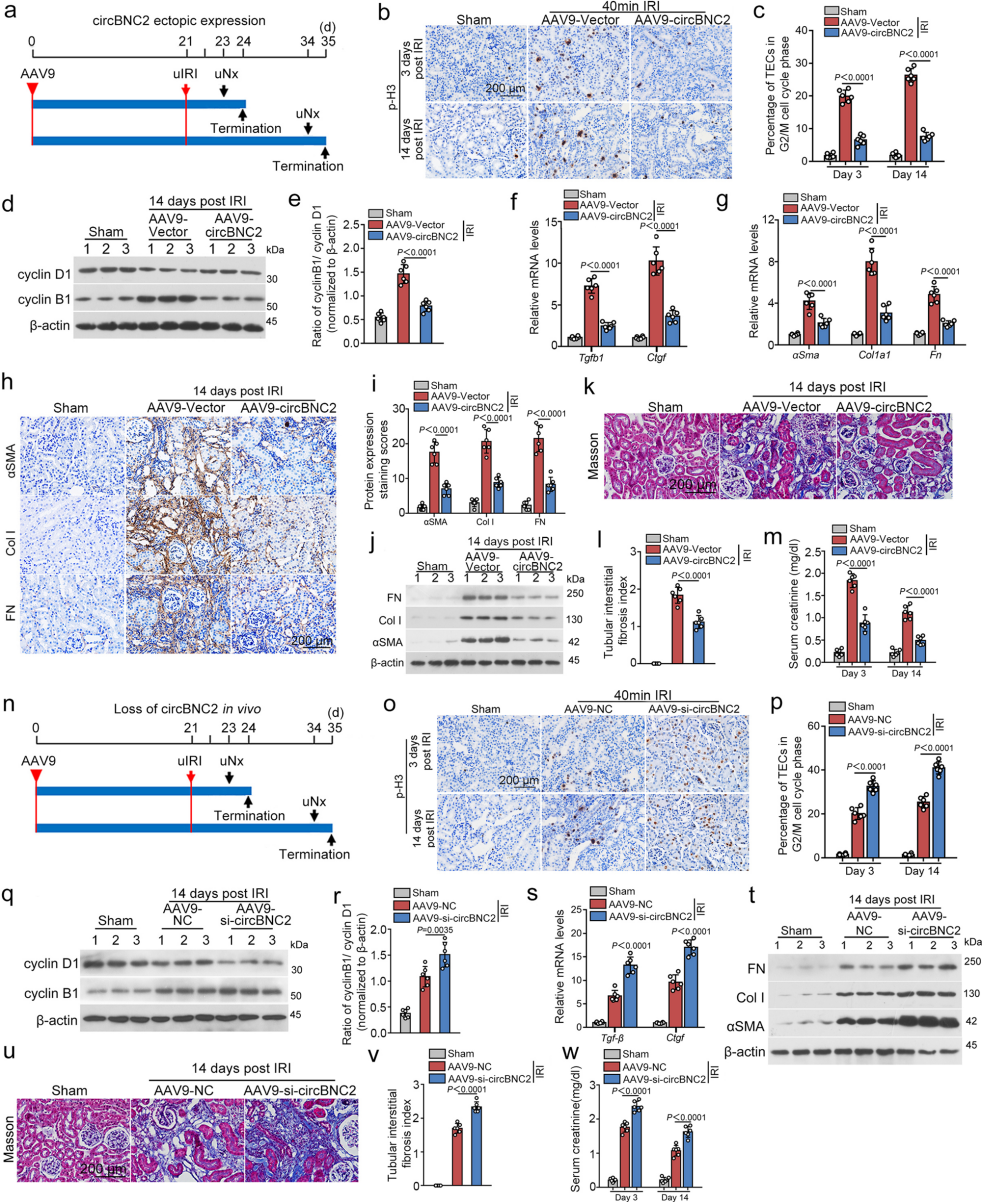
circBNC2 alleviates fibrotic maladaptive repair in kidney after injury. **a** Mice were treated by a single-dose left renal vein injection of either AAV9-circBNC2 or AAV9-Vector at 21 days before processing unilateral IRI (on the left side). Animals were euthanized 3 or 14 days after processing IRI. **b**, **c** Immunohistochemistry showing p-H3 staining in kidney sections from mice delivered with circBNC2 and subjected to IRI (**b**), and the quantification of p-H3 positive cells (**c**). **d**, **e** Western blots showing the expression of cyclin B1 and cyclin D1 in the renal cortex homogenates from mice delivered with circBNC2 and subjected to IRI (**d**), and the quantification of cyclin B1/cyclin D1 (**e**). **f**, **g** qRT-PCR showing the expression of profibrotic factors in kidneys from mice treated with AAV9-circBNC2 delivery and IRI. **h**, **i** Immunohistochemistry showing αSMA, Col I and FN deposition was decreased in kidney sections from mice treated with AAV9-circBNC2 delivery and IRI (**h**), and the quantification data (**i**). **j** Western blots showing αSMA, Col I and FN expression in kidney homogenates from IRI mice overexpressed with circBNC2. **k**, **l** Masson staining of kidney sections from IRI mice treated with AAV9-circBNC2 or AAV9-Vector (**k**), and the quantification data (**l**). **m** Delivery of circBNC2 in IRI mice reduced the levels of serum creatinine at day 3 and 14 after IRI compared with the IRI mice injected with AAV9-Vector. **n** Mice were treated by left renal vein injection of either AAV9-si-circBNC2 or AAV9-NC 21 days before processing unilateral IRI (on the left side). Mice were euthanized 3 or 14 days after processing IRI. **o**, **p** Immunohistochemistry showing p-H3 staining in kidney sections from mice delivered with si-circBNC2 and subjected to IRI (**o**), and the quantification of p-H3-positive cells (**p**). **q**, **r** Western blots showing cyclin B1 and cyclin D1 expression in renal crotex homogenates from IRI mice administrated with AAV9-si-circBNC2 or AAV9-NC at day 14 post IRI (**q**), and the quantification data (**r**). **s** qRT-PCR showing the expression of *Tgfb1* and *Ctgf* in kidneys from mice with circBNC2 knockdown and subjected to IRI. **t** Western blots showing expression of ECM in kidneys from IRI mice administrated with AAV9-si-circBNC2 or AAV9-NC. **u**, **v** Masson staining of kidney sections from IRI mice administrated with AAV9-si-circBNC2 or AAV9-NC (**u**), and the quantification data (**v**). **w** Administration of si-circBNC2 in IRI mice increased the levels of serum creatinine at day 3 and 14 after IRI compared with the IRI mice treated with AAV9-NC. For **c**, **e**–**g**, **i**, **l**, **m**, **p**, **r**, **s**, **v**, **w**, *n* = 6 in each group. Data are expressed as means ± SD. One-way ANOVA with Bonferroni post hoc test was used for comparison among multiple groups (**c**, **e**, **f**, **g, i**, **l**, **m**, **p**, **r**, **s**, **v**, **w**). Source data are provided as a Source Data file.

In a unilateral IRI (40-min ischemia)-induced renal fibrosis model, mice were treated with adeno-associated virus serotype 9 (AAV9)-carrying circBNC2 three weeks before operation. Animals were euthanized at day 3 or 14 after ischemia (Fig. 5a). Northern blots of kidney homogenates and in situ hybridization in renal tissue demonstrated that AAV9-circBNC2 treatment increased circBNC2 expression in injured kidneys (Supplementary Fig. 5j–l). IRI mice bearing circBNC2 presented decreased numbers of p-H3-stained cells in their kidneys (Fig. 5b, c) and diminished ratios of cyclin B1/cyclin D1 (Fig. 5d, e), as compared with mice treated with the empty AAV9 vector. Furthermore, delivery of circBNC2 decreased expression of *Tgfb1* and *Ctgf* in renal cortex homogenates (Fig. 5f), diminished ECM accumulation in renal tissue (Fig. 5g–j), attenuated kidney fibrosis (Fig. 5k, l), and improved renal function (Fig. 5m). The expression and amounts of AAV9-carrying circBNC2 in the kidneys and other organs were determined by co-expressing a luciferase tag with exogenic circBNC2. The luminescent signals were mainly located in the left kidney when mice were treated with AAV9-circBNC2 through the left renal vein. In contrast, in mice treated with AAV9-circBNC2 through the tail vein, the luminescent signals were located in the liver and bilateral kidneys (Supplementary Fig. 5m–o). Furthermore, immunofluorescence assays following fluorescence in situ hybridization assays demonstrated the entry of exogenic circBNC2 into kidney cells, as reflected by the co-localization of exogenic FLAG tags with circBNC2 (Supplementary Fig. 5p).

In an AA-induced kidney injury model, mice were delivered with circBNC2 three weeks before the injection of AA and euthanized 8 weeks after AA exposure (Supplementary Fig. 6a). The delivery treatment effectively increased the expression of circBNC2 in the kidneys (Supplementary Fig. 6b, c). Delivering circBNC2, decreased cell G2/M arrest frequency as evidenced by decreased p-H3 stained cell numbers and reduced ratios of cyclin B1/cyclin D1 (Supplementary Fig. 6d–g), decreased expression of *Tgfb1* and *Ctgf* (Supplementary Fig. 6h), attenuated ECM accumulation (Supplementary Fig. 6i, j), attenuated ECM accumulation, ameliorated renal fibrosis (Supplementary Fig. 6k, l), and improved renal function, as compared to that in aristolochic acid nephropathy mice treated with AAV9-Vector.

To perform loss-of-function studies in vivo, mice were delivered with AAV9-carrying siRNA targeting circBNC2 three weeks before severe IRI treatment and euthanized at day 3 or 14 post ischemia (Fig. 5n). Knocking-down circBNC2 with siRNAs significantly decreased the expression of circBNC2 in the kidneys (Supplementary Fig. 6n–p). Compared with that in IRI mice treated with AAV9-NC, loss-of-function in circBNC2 resulted in further-increased p-H3 positive cell abundance in renal sections (Fig. 5o, p), enhanced the ratio of cyclin B1/cyclin D1 (Fig. 5q, r), increased the expression of profibrotic factors (Fig. 5s, t), elevated ECM deposition in the kidney cortex (Fig. 5u, v), and further deteriorated renal function in IRI mice (Fig. 5w).

### Overexpression of circBNC2 inhibits liver fibrosis by attenuating hepatocytes G2/M arrest

**Fig. 6:**
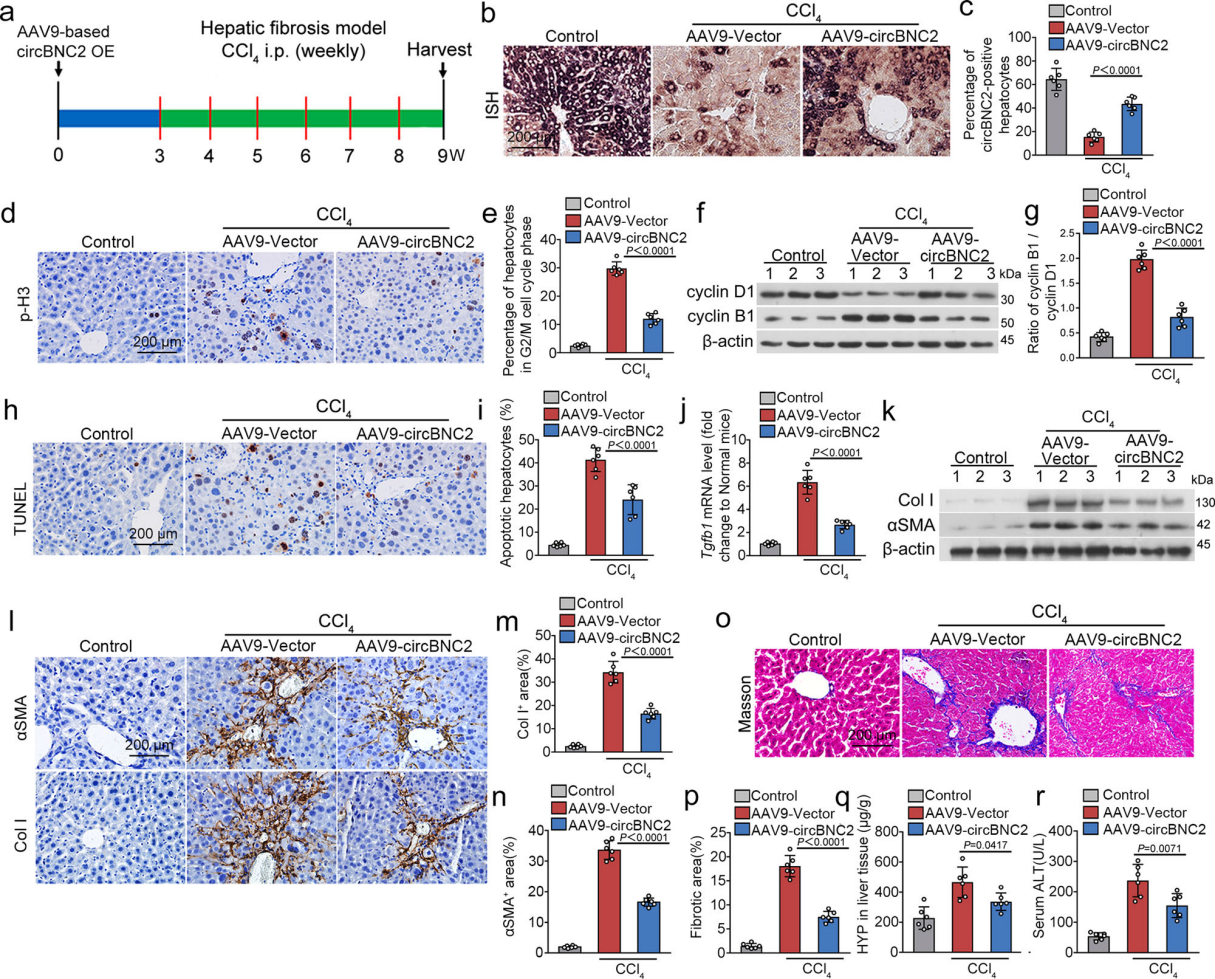
Overexpression of circBNC2 inhibits liver fibrosis by attenuating hepatocytes arrested in G2/M phase. **a** Mice were treated by tail vein injection of either AAV9-circBNC2 or AAV9-Vector 3 weeks before processing hepatic fibrosis model by injecting CCl_4_ (once a week for 6 weeks). Mice were euthanized 6 weeks post the first injection of CCl_4_. **b**, **c** Representative images of in situ hybridization of circBNC2 expression in livers from mice injected with AAV9-circBNC2 (**b**) and the quantification data (**c**). **d**, **e** Representative images of Immunohistochemistry of p-H3 in liver sections from mice treated with AAV9-circBNC2 and CCl_4_ (**d**), and the quantification data (**e**). **f**, **g** Western blots showing expression of cyclin B1 and cyclin D1 in mice treated with AAV9-circBNC2 and CCl_4_ (**f**), and the quantification data (**g**). **h**, **i** Representative images of TUNEL staining of apoptotic cells in the sections from CCl_4_-treated mice overexpressed with circBNC2 (**h**), and the quantification data (**i**). **j** qRT-PCR showing the expression of *Tgfb1* in livers from CCl_4_-treated and circBNC2-overexpressed mice. **k** Western blots showing expression of αSMA and Col I in the liver homogenates from CCl_4_-injected mice overexpressed with circBNC2. **l**–**n** Representative images of Immunohistochemistry of αSMA and Col I in liver sections from circBNC2-overexpressed mice treated with CCl_4_ (**l**) and the semi-quantification data (**m**, **n**). **o**, **p** Representative images of Masson staining of liver sections from CCl_4_-injected mice with circBNC2 overexpression (**o**), and the quantification data (**p**). **q** Reduced hydroxyproline (HYP) in liver tissues in CCl_4_-injected mice with circBNC2 overexpression. **r** Overexpression of circBNC2 improved liver function in CCl_4_-injected mice, evidenced by decrease in serum alanine aminotransferase (ALT). For **c**, **e**, **i**, **j**, **m**, **n**, **p**–**r**, *n* = 6 in each group. Data are expressed as means ± SD. One-way ANOVA with Bonferroni post hoc test was used for comparison among multiple groups (**c**, **e**, **g, i**, **j**, **m**, **n**, **p**, **q**, **r**). Source data are provided as a Source Data file.

In a CCl_4_-induced liver fibrosis model, mice were treated with AAV9-circBNC2 delivered through the tail vein 3 weeks prior to injection of CCl_4_ and euthanized 6 weeks thereafter (Fig. 6a). Ectopic expression of circBNC2 significantly increased circBNC2 expression in hepatocytes compared to that of mice treated with the empty AAV9 vector (Fig. 6b, c). Compared to CCl_4_-treated mice administered the AAV9 vector only, delivery of circBNC2 decreased hepatocyte G2/M arrest in the liver, as demonstrated by decreased p-H3-positive cells and cyclin B1/cyclin D1 ratios (Fig. 6d–g). Overexpression of circBNC2 attenuated CCl_4_-induced hepatocyte apoptosis (Fig. 6h, i), decreased the hepatic expression of *Tgfb1* (Fig. 6j), and ameliorated expression of αSMA and Col I in the injured liver (Fig. 6k–n). Furthermore, hepatic fibrosis was attenuated (Fig. 6o–q) and liver function was improved (Fig. 6r) by ectopic expression of circBNC2.

In addition, compared to CCl_4_-treated mice delivered with the empty vector, ectopic expression of circBNC2 in CCl_4_-treated mice reduced the mRNA levels of profibrotic factors such as *Tgfb1*, *Col1a1* and *Timp-1*, and increased the mRNA levels of *Mmp-13* at days 3, 6 and 8 after a single treatment of CCl_4_ (Supplementary Fig. 7a–e).

### Downregulation of circBNC2 and ctBNC2 associates with epithelial cell G2/M arrest in human fibrotic kidney and liver tissue

**Fig. 7:**
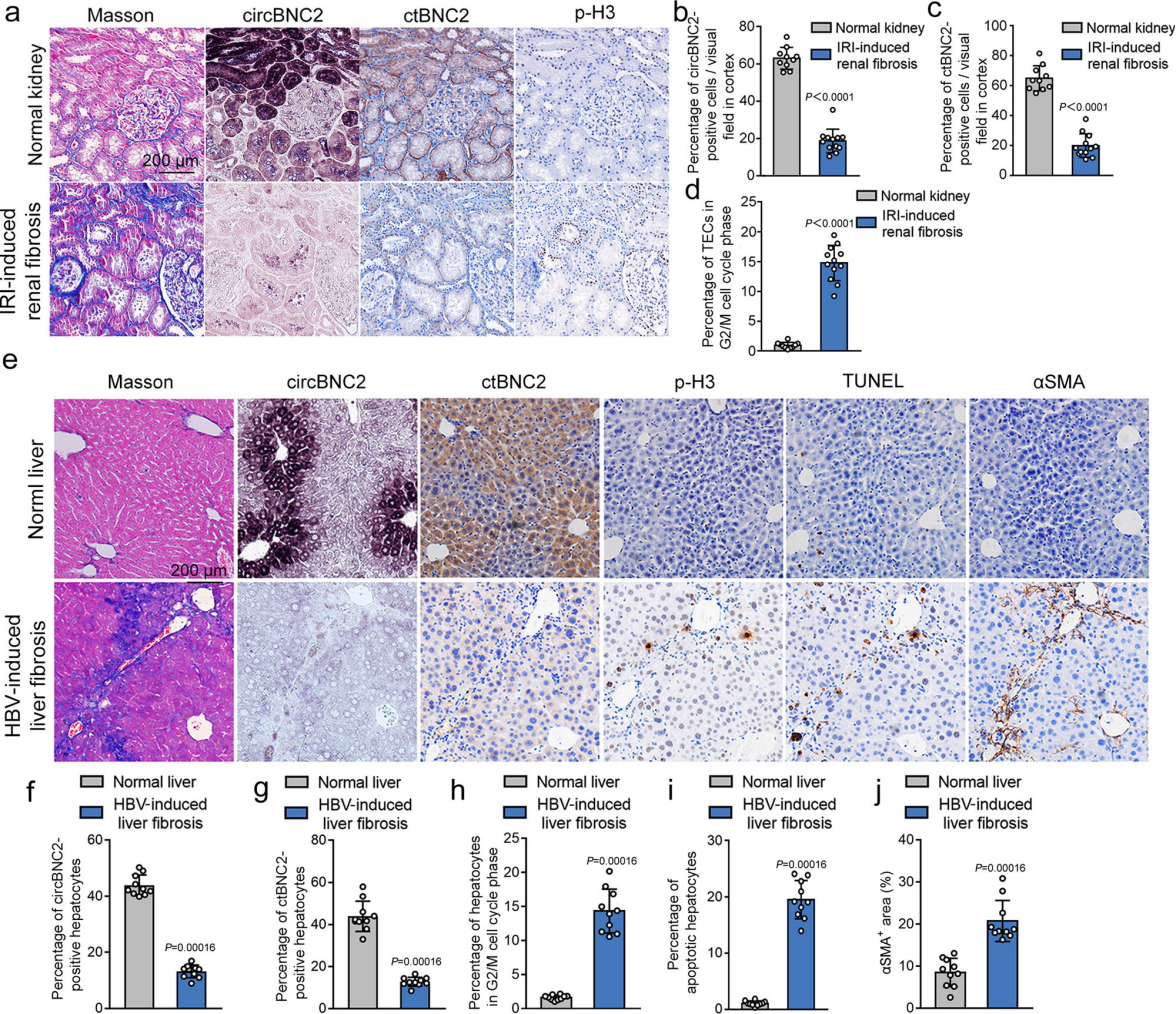
Downregulation of circBNC2 and ctBNC2 associates with epithelial cell G2/M arrest in human fibrotic kidney and liver. **a**–**d** Representative images showing Masson staining, circBNC2 and ctBNC2 expression, and expression of p-H3 in serial sections of normal kidney and IRI-induced kidney fibrosis (**a**) and the semi-quantitative data (**b**–**d**). For **b**–**d**, *n* = 10 for control, *n* = 12 for patients with IRI-induced kidney fibrosis). **e**–**j** Representative photos showing Masson staining, circBNC2 and ctBNC2 expression, expression of p-H3, TUNEL positive cells, and levels of αSMA, in serial sections of normal liver and hepatitis B virus (HBV)-induced liver fibrosis (**e**) and the semi-quantitative data (**f**–**j**). For **f**–**j**, *n* = 10 tissue in each group. Data are expressed as means ± SD. Two-sided Mann–Whitney *U* test was used for the comparison of two groups (**b**, **c**, **d**, **f**, **g**, **h**, **i**, **j**). Source data are provided as a Source Data file.

To validate the results in human fibrotic diseases, serial sections of kidney and liver tissue biopsies were used to analyze the association of circBNC2 and ctBNC2 with fibrotic maladaptive repair (Fig. 7). Downregulation of circBNC2 and ctBNC2 was observed in human ischemic fibrotic kidney, which was also associated with increased epithelial cell G2/M arrest, as manifested by elevated numbers of p-H3-stained cells (Fig. 7a–d). Similarly, expression of circBNC2 and ctBNC2 was downregulated in samples of liver fibrosis induced by the hepatitis B virus, which was associated with increased p-H3-stained hepatocytes, apoptotic hepatocytes, and activated stellate cells (Fig. 7e–j). Downregulation of circBNC2 was associated with fibrosis in both the kidney and liver (Fig. 7a, e).

## Discussion

The kidneys and liver are the organs most highly exposed to toxic or ischemic insults^1,2^. Cellular maladaptive repair occurs during the early phase of profibrotic responses and plays a critical role in the pathogenesis of subsequent organ fibrosis. Thus, unveiling the mechanisms underlying maladaptive repair is of particular importance to develop therapeutic interventions against the progression of organ fibrosis. In this study, we identified a kidney- and liver-enriched circular RNA, circBNC2, which was abundantly expressed in normal TECs and hepatocytes and downregulated in cells after acute ischemic or toxic insults. We found that the downregulated expression of circBNC2 was at least partially mediated by the upregulation of DHX9, an abundant nuclear RNA helicase^23^. However, further studies are required to demonstrate how DHX9 upregulation can lead to the downregulation of circBNC2 levels within the experimental timeframes described here. Similarly, in a previous study^13^, it was reported that circRNA SCAR is downregulated within 24 h post-palmitate stimulation. Here, circBNC2 acted as an endogenous negative regulator of cell cycle G2/M arrest through encoding ctBNC2, a previously undiscovered protein, that promoted the formation of CDK1/cyclin B1 complexes, which specifically regulated cellular entry into mitosis^25^. To our knowledge, this is the first study to define the functionality of circBNC2 and highlight the biological significance of circRNA in regulating the cell cycle in normal epithelial cells. More importantly, we demonstrated that restoring circBNC2 improved kidney and liver fibrotic maladaptation and attenuated fibrosis, suggesting that it might represent a potential strategy for therapeutic intervention in epithelial organ fibrosis.

circBNC2 was originally identified in myoblasts^26^. Our study examined the expression of circBNC2 in various tissues and demonstrated that circBNC2 was most abundant in the kidney and, to a lesser extent, in the liver. By combining several experimentally induced kidney and liver fibrosis models in mice, in vitro studies with epithelial cells, unbiased gene expression analyses and validation in human biopsies, we characterized circBNC2 as a negative regulator of epithelial cell cycle arrest, a key checkpoint for directing the wound-healing process toward either normal cell proliferation and differentiation or fibrotic maladaptive repair^27,28^. In our study, knock-out of circBNC2 in normal human TECs or hepatocytes significantly increased the number of cells arrested in the G2/M phase. These cells participated in maladaptive repair by producing profibrotic factors such as TGF-β and CTGF, which are capable of activating fibroblasts or stellate cells and stimulating ECM production and accumulation^29–31^. Furthermore, in experimental models of kidney and liver fibrosis, the expression of circBNC2 was downregulated and associated with an increased frequency of p-H3-positive cells, a marker of G2/M-phase cells. Restoring the expression of circBNC2 in the experimental models of fibrosis significantly decreased ECM deposition and alleviated fibrotic lesions in diseased kidneys and liver. Moreover, we confirmed that these findings were recapitulated in human IRI-induced chronic kidney disease and inflammation-induced liver disease, underscoring the clinical relevance.

Non-coding RNAs such as miRNAs, lncRNAs and circRNAs, constitute >90% of all RNAs transcribed from the human genome. However, their functions remain largely unknown and await elucidation^32^. Although circRNAs are generally considered to be non-coding, several studies have provided evidence that certain circRNAs can be translated into proteins^18,26,33^. We found that circBNC2 contained an IRES sequence that could actively induce 5’-cap-independent translation. Employing CPC^24^, circBNC2 was predicted to be translated into a protein containing 681 aa. Using MS, we identified a unique peptide sequence translated from circBNC2 junction sequences. By using a specific antibody designed to recognize this unique peptide sequence, we identified a previously unrecognized protein with a molecular weight of 75 kDa, which was in accord with the size of a protein composed of 681 aa in TECs and hepatocytes. We designated this as ctBNC2 and further verified its functions. The functional significance of many circRNA-derived proteins remains a mystery, although some have been implicated in carcinogenesis^18^. CDK1 binding to cyclin B1 and then translocating into cell nuclei have been recognized as critical steps for the initiation of mitosis^19,20^. Evidence from in vitro studies involving TECs and hepatocytes show that ctBNC2 promotes G2 to M cell cycle progression by interacting with CDK1 and cyclin B1 to form a ternary complex, which is translocated into the nuclei. The regulatory effects of circBNC2 on G2/M cell cycle arrest may be specifically induced by ctBNC2 and also depend on both CDK1 and cyclin B1, since overexpressing BNC2-FL had no effect on G2/M cell cycle arrest or CDK1-cyclin B1 complex formation in injured TECs. These data might suggest that circBNC2 protects injured epithelial cells from G2/M cell cycle arrest by encoding a protein, ctBNC2, that mediates CDK1/cyclin B1 complex formation.

**Fig. 8:**
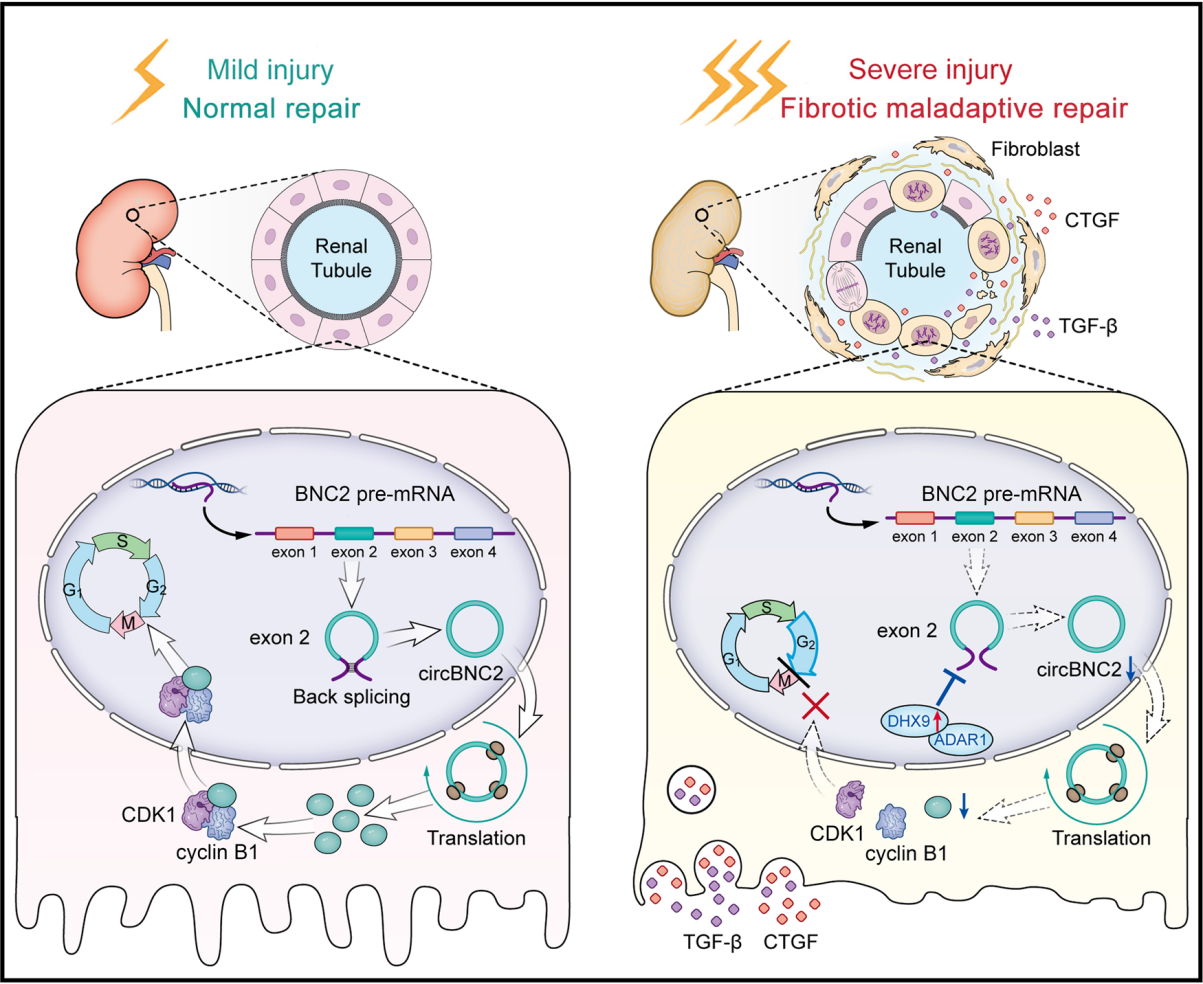
Proposed model for cell cycle regulation by circBNC2. A protein encoded by circBNC2 is dysregulated in severe injured epithelial cells, resulting in the G2/M cell cycle arrest and the subsequent fibrotic maladaptive repair.

In conclusion, we demonstrated that circBNC2 is a key regulator of G2/M arrest in TECs and hepatocytes in normal wound-healing. Downregulation of circBNC2 after severe injury facilitated fibrotic maladaptive repair by increasing epithelial cell G2/M arrest, thereby promoting fibrosis progression (Fig. 8). These findings suggest that preventing fibrogenic responses by regulating cell cycle arrest may be a potential therapeutic strategy in organ fibrosis.

## Methods

### Cell line and primary cell isolation

The following cell lines used in this study were commercially available: the human tubular epithelial cell line HK2 [American Type Culture Collection (ATCC)], the human hepatocyte cell line L-02 (Shanghai Cell Bank, Shanghai, China), human embryonic kidney (HEK)−293T cells (Shanghai Cell Bank), human mesangial cells (ScienCell) and the human hepatic stellate cell line LX-2 (Merck Millipore). Mouse mesangial cells (sv40mes13) (Cellcook, Guangzhou, China).

Mouse immortalized tubule epithelial cell (mTEC) was a gift from H.-Y. Lan (The Chinese University of Hong Kong). HKC8 cells were a gift from L. Racusen (Johns Hopkins University). The conditionally immortalized mouse podocyte cell line (MPC-5) was provided by Peter Mundel (Massachusetts General Hospital, Boston, MA).

Primary mouse hepatic stellate cells and hepatocytes were isolated from mice by two-step collagenase perfusion method^34^. Briefly, liver tissue was perfused with 0.05% pronase E (Roche) and 0.03% collagenase type IV (Sigma-Aldrich) through the portal vein until the liver became pale in color and then liver was further digested with collagenase type IV, pronase E and DNase I (Roche) solution at 37 °C bath shaking for 30 min. The suspension was filtered through a 100-μm pore size mesh nylon filter (Sinopharm), and then centrifuged for 10 min at 300 × g, the pellet was collected for primary hepatocytes and the supernate for primary hepatic stellate cells. Cells were cultured in RPMI 1640 medium supplemented with 10% fetal bovine serum (Invitrogen)^35^.

### Cell culture

HK2 cells, HKC8 cells, sv40mes13 cells and mTEC cells were grown in Dulbecco’s modified Eagle’s medium (DMEM)/Ham’s F12 medium (Invitrogen) supplemented with 10% fetal bovine serum (FBS) (Invitrogen). HMC cells, L-02 cells and LX-2 cells were grown in RPMI 1640 (Invitrogen) supplemented with 10% FBS. HEK-293T cells were cultured in DMEM (Invitrogen) supplemented with 10% FBS. These cells were cultured in an incubator (Thermo Fisher Scientific) under normoxic condition (21% O_2_, 5% CO_2_, 37 °C). The human podocytes were cultured under permissive condition in RPMI 1640 media (Invitrogen) containing 10% FBS, 0.1% antibiotic/antimycotic solution, and Insulin-Transferrin-Selenium-Ethanolamine solution (ITS) (Invitrogen) at 33 °C in an incubator with 5% CO_2_. When podocytes reached 70% confluence, they were cultured at 37 °C for another 8 days to induce cell differentiation. To propagate mouse podocytes (MPC-5), cells were cultured at 33 °C in RPMI 1640 media supplemented with 10% FBS and 10 U/ml mouse recombinant interferon gamma (IFN-γ; R&D Systems) to enhance the expression of a thermos-sensitive T antigen. To induce differentiation, podocytes were grown under nonpermissive conditions at 37 °C in the absence of IFN-γ.

To perform Aristolochic Acid (AA) treatment in cultured cells, cells were deprived of serum overnight when they reached about 70% confluence and then incubated with AA (Sigma-Aldrich) for the indicated timepoints. To establish hypoxia model, cells were deprived of serum overnight when they reached about 70% confluence. Then the cells were incubated in low-glucose DMEM (Invitrogen) at 37 °C for the indicated timepoints in a hypoxia chamber (1 % O_2_, 5% CO_2_, 94% N_2_). In some experiments, LX-2 cells were treated with conditional medium from wild-type or circBNC2-KO L-02 cells for the indicated timepoints.

### Animal studies

#### Mice and animal housing

Male C57BL/6, aged 6–8 weeks (20–24 g), purchased from Guangdong Medical Animal Centre were used to establish kidney or liver injury model in accordance with the institutional guidelines of Southern Medical University. Mice were housed in a standard environment which was characterized by 12 h light/dark cycle, 22–25 °C and 40–60% humidity with free access to water and forage. All animal studies were approved by the Nanfang Hospital Animal Care Committee. Animal welfare was reviewed during the application of the animal experiments by the Nanfang Hospital Animal Care Committee, and was monitored during the experiments by the employees of the Laboratory Animal Center of Nanfang Hospital. To euthanatize the mice before collecting tissue and blood samples, the mice were placed in a plexiglass chamber with 5% isoflurane (RWD, Cat #R510-22, Shenzhen) for 5 min. After that, cervical dislocation was performed when mice were fully sedated, as measured by a lack of active paw reflex.

#### Models of kidney injury

Briefly, mice were anesthetized by sodium pentobarbital. Ischemia-reperfusion injury (IRI) was induced by clipping bilateral or the left renal pedicle for 20-min (mild AKI) or 40-min (severe AKI) by using the microaneurysm clamps (Fine Science Tools company, Cat #18051-35, Cambridge, UK). The body temperature of the animals was maintained between 37 and 38 °C by applying a temperature-controlled heating device. For mice subjected with unilateral IRI model (left-side kidney), 2 or 13 days after unilateral IRI, the right-side kidney was surgically removed to evaluate the kidney function of the left-side kidney. Sham operations were performed with exposure of both kidneys but without ischemia treatment. To minimize the variations caused by surgery, all mice surgery was carried out by the same investigator under the identical conditions.

AA-induced kidney injury was prepared by a single-dose intraperitoneal injection of AA (5 mg per kg weight) (Sigma-Aldrich) in PBS. The mice in control group were injected intraperitoneally with the same amount of PBS. Mice were sacrificed 8 weeks after the AA injection.

#### Models of acute liver injury and liver fibrosis

To evaluate the function of circBNC2 on fibrosis and fibrolysis in the repair process following acute liver injury, mice were intraperitoneally injected with a single dose of carbon tetrachloride (CCl_4_) (1 ml/kg, CCl_4_ dissolved in olive oil at a ratio of 1:4). Then the mice were sacrificed at day 3, 6 and 8 after CCl_4_ injection. Mouse liver fibrosis model was established by intraperitoneally injection of CCl_4_ (1 ml per kg, CCl_4_ dissolved in olive oil at a ratio of 1:4) weekly for 6 weeks. The mice in Control group were treated with the same amount of olive oil only.

### RNA quantification

#### High-throughput RNA-sequencing for circRNA

To identify the differentially expressed circRNAs in renal tubules of Sham and IRI mice, the renal cortex was dissected and digested in 1 mg/ml collagenase type IV (Sigma-Aldrich) for 30 min at 37 °C, and then were resuspended and washed twice with cold PBS. The tubules were isolated using 31% Percoll gradients (General Electric), then resuspended, and washed with cold PBS. Total RNAs of the collected tubules were extracted with TRIZOL reagent (Invitrogen).

For the preparation of RNA-sequencing library, total RNAs (3 μg) from each sample were treated with the VAHTS Total RNA-sequencing (H/M/R) Library Prep Kit for Illumina (Vazyme Biotech, Nanjing, China) to erase ribosomal RNA but retained mRNA and non-coding RNA (ncRNA). RNA-sequencing libraries were constructed using the KAPA Stranded RNA-sequencing Library Prep Kit (Roche) and subjected to deep sequencing with an Illumina HiSeq 4000 (Aksomics, Shanghai). To evaluate the expression of circRNA after RNA-sequencing, the junction site was identified by STAR software, then the backsplice junction reads were calculated by CIRCexplorer2. circRNAs were annotated through blasting the sequence in circBase^36^. To identify differentially expressed circRNAs across samples, the edgeR package (http://www.r-project.org/) was used. We chose circRNAs with a fold change ≥1.5 and a *P* value <0.05 in a comparison between IRI and Sham mouse renal tubules as significantly differentially expressed circRNAs.

#### mRNA sequencing and functional enrichment analysis

To reveal the functional enrichment analysis of the differentially expressed mRNAs between circBNC2-KO and wild-type cells. Total RNAs were extracted and measured for the quality before the sequencing libraries were constructed with KAPA stranded RNA-sequencing Library Prep Kit (Roche). The RNA-sequencing libraries were sequenced with an Illumina X-ten/NovaSeq (Aksomics). Sequencing reads were filtered using software FastQC. Clean reads were aligned to the human reference genome GRCh37 (NCBI) using HISAT2 (Hierarchical Indexing for Spliced Alignment of Transcripts), and gene expression level was then calculated using the RSEM software package. To identify differentially expressed mRNAs across samples, the edgeR package was used. We chose mRNAs with a fold change ≥2 and a *P* value <0.05 in a comparison between circBNC2-KO and wild-type cells as significant differentially expressed mRNAs. The differentially expressed mRNAs were analyzed with Database for Annotation Visualization and Integrated Discovery (DAVID) for Gene Ontology (GO) enrichment analysis. The enriched biological process (BP) that had *P* < 0.05 was considered significantly enriched.

#### Real-time quantitative RT-PCR (qRT-PCR)

Total RNAs were isolated from tissue and cultured cells using TRIzol reagent (Invitrogen, Carlsbad, CA, USA) according to the manufacturer’s instructions. After testing the quality and integrity of RNAs, first-strand cDNA was prepared using the HiScript® II 1st Strand cDNA Synthesis Kit (Vazyme, R211) and quantitative RT-PCR was performed using ChamQ Universal SYBR qPCR Master Mix (Vazyme, Q711) according to the manufacturer’s instructions, separately. In particular, to determine the abundance of circRNAs, the divergent primers were designed to amplify the products containing the specific junction site. Sequences of the primer pairs were listed in Supplementary Table 2. The PCR efficiencies of interest and the reference genes (The MIQE checklist^37^) were listed in Supplementary Data 3. All the PCR efficiencies were between 91.9 and 107%. For relative mRNA or circRNA expression, the target gene expression was calculated as 2^−ΔCt^ (Ratio of specific mRNAs or circRNAs to β-actin mRNA) or calculated using the comparative 2^−ΔΔCt^ method. To determine the cellular location of circBNC2, the nuclear and cytoplasmic fractions of HK2 cells were isolated with PARIS Kit (Thermo Fisher Scientific) according to the manufacturer’s instructions. Total RNAs extraction and qRT-PCR were conducted in nuclear and cytoplasmic fractions, separately.

#### Northern blot analysis

Northern blots were performed as previously described^38^. Briefly, total RNAs were separated on an agarose (1.5%)-formaldehyde (2.2 M) gel. After being transmembraned and crosslinked, the nylon membranes (Solarbio, Beijing, China) were incubated with 5’ digoxin labeled LNA-modified probes targeting both circBNC2 and BNC2 mRNA or specific to circBNC2 or β-actin mRNA separately at 55 °C overnight. The membranes were adequately washed in 2 × SSC buffer (Boster, Wuhan, China) at room temperature and incubated with anti-DIG conjugate followed by visualizing with the chemiluminescence substrate CSPD supplied in DIG Luminescent Detection Kit (Roche) according to the manufacturer’s instructions. The probes were synthesized by Exiqon according to the sequences of the target genes listed in Supplementary Table 2.

To determine the stability of circBNC2, total RNAs (2.5 μg) from HK2 cells was degraded for 20 min at 37 °C with or without 5 U/μg RNase-R (Epicenter Technologies), followed by northern blots. To test the stability of circBNC2, HK2 cells were seeded in six-well plates and treated with 100 ng/ml actinomycin D (Amresco) to inhibit new RNA synthesis for 8, 16 and 24 h. Then, circBNC2 and BNC2 mRNA were detected by qRT-PCR.

### Cell cycle analysis

#### Flow cytometry analysis

Flow cytometry was performed using a BD Analyzer (BD Biosciences). For cell cycle assay, HK2 cells and L-02 cells were trypsinized, centrifuged and fixed with 70% ice cold ethanol and stored at −20 °C. Twenty-four hours later, the fixed cells were stained with propidium iodine (PI) staining solution (Lianke, Hangzhou, China). To quantify the percentage of M phase cells, the fixed cells were permeabilized with 0.3% Triton X-100 and incubated with anti-p-H3 (Ser 10) primary antibody (Cell Signalling Technology, cat. 53348, dilution 1:800) for 30 min and then stained with secondary antibody conjugated with Alexa Fluor 647 (Cell Signalling Technology, cat. 4414, dilution 1:500) for 30 min before PI staining. The representative gating strategy for this experiment was presented in Supplementary Fig. 8a.

#### Immunofluorescence staining for Phospho-Histone H3 (Ser 10)

Immunofluorescence staining of formaldehyde-fixed cells was performed as previously described^38^. Briefly, the cells were washed with cold PBS, fixed in 4% formaldehyde, permeabilized with 0.5% Triton X-100, and incubated with the specific primary antibody against p-H3 (Ser 10) (Cell Signalling Technology, cat. 53348, dilution 1:200) at 4 °C, overnight. Cells were incubated with secondary antibody conjugated with Alexa Fluor 488 (Cell Signalling Technology, cat. 4412, dilution 1:200) for 1 h at room temperature. Actin filaments were labeled with DyLight 594 Phalloidin (Cell Signalling Technology, cat. 12877, dilution 1:100) The nuclei were visualized by staining with 4’,6-diamidino-2-phenylindole (DAPI, C1006, Beyotime). Images were taken using a confocal microscopy (Leica TCS SP8, Leica Microsystems).

### Cell apoptosis assays

For apoptosis assay, cells were stained with annexin V and PI for 30 min with the FITC Annexin V Apoptosis Detection Kit II (BD Biosciences) according to the manufacturer’s instructions. Data analysis was performed using FlowJo and Modfit software. The representative gating strategy for this experiment was presented in Supplementary Fig. 8b.

### Gene gain-of-function and loss-of-function analysis in vitro

#### circBNC2 overexpression

The full-length sequence or ATG-deleted sequence of circBNC2 was separately inserted into pLO5-ciR (Geneseed Biotech, China) at the EcoRI and BamHI sites. The lentivirus was produced in HEK-293T cells with the packaging plasmids. Infectious lentivirus was harvested 60 h after transfection and filtered through 0.45-μm filters (Merck Millipore). The cell lines were infected with these lentivirus for 24 h, then the cells were selected with 2 μg/ml puromycin for a week to select positive clones. To overexpress circBNC2 in a dose-dependent manner, the full-length sequence of circBNC2 was cloned into pcD-ciR (Geneseed Biotech) for transient transfection into HK2 cells. Three doses (0.5, 1 and 2 μg plasmids per 1 × 10^7^ cells) of pcD-circBNC2 plasmids were used to transfect HK2 cells using lipofectamine 3000 according to the manufacturer’s instructions (Invitrogen).

#### BNC2-FL and ctBNC2 overexpression

For transient transfection, the coding sequence of BNC2-FL (1099 amino acids) or ctBNC2 (681 amino acids) was tagged with FLAG and cloned into pcDNA3.1 vector (Invitrogen), separately. Cells were transfected with pcDNA3.1-BNC2-FL or pcDNA3.1-ctBNC2 for 24 h with or without treating cells with hypoxia for the indicated timepoints. To overexpress ctBNC2 in a dose-dependent manner, three doses (1, 2 and 4 μg plasmids per 1 × 10^7^ cells) of ctBNC2 plasmids were transfected into HK2 cells using lipofectamine 3000.

#### circBNC2 knockout

To investigate the function of circBNC2, we took advantage of CRISPR-Cas9 mediated gene editing. The small guide RNAs targeting the two introns beside exon 2 of BNC2 were designed for knocking out circBNC2 while reserving the protein expression of lBNC2. To establish circBNC2 knockout cell line, four small guide RNAs (sgRNAs) targeting the introns on both sides of exon 2 of lBNC2 were cloned into CV279 plasmid. The sequences of relative sgRNAs were listed as follows, sgRNA1: ACATACTGCACGGCCTTGGC, sgRNA2: ACTCTGCGGGACTATGTCCG, sgRNA3: GAGTTGACTCTGTCAATGGT, sgRNA4: GTGGTGAGAGGTGCAATCAG. The lentivirus was produced by Genechem (Shanghai, China) and applied to infect HK2 cells and L-02 cells according to the manufacturer’s instructions. The cells were selected with 2 μg/ml puromycin for a week to select positive clones.

#### lBNC2 knockout

We used CRISPR-Cas9 systems with the small guide (sg)-RNA targeting Exon 1 of BNC2 gene to induce frameshift mutation of BNC2 protein (loss of BNC2 protein expression) without affecting the expression of circBNC2. The sgRNA sequences were listed as follows: CGCTGAAGAGACGGTCCAGC. The lentivirus was produced by Genechem and applied to infect HK2 cells according to the manufacturer’s instructions. The positive clones were selected by 2 μg/ml puromycin for a week.

#### RNA interference

For transient transfection, 50–80% confluent cells were transfected with the siRNAs using lipofectamine 3000 according to the manufacturer’s instructions (Invitrogen). Target sequences of siRNAs for DHX9, ADAR1, CDK1 and cyclin B1 are listed in Supplementary Table 2.

### Open reading frame (ORF) prediction and verification

To predict ORF in circBNC2, we used the Coding Potential Calculator (CPC)^24^ as previously described^13^. The full-length circBNC2 sequences were sent to the CPC website for coding potential predictions based on the default parameters.

To test whether the predicted ORF sequence in the circBNC2 could be translated into protein, we inserted a FLAG-coding sequence into circBNC2 overexpression vector. Briefly, circBNC2–3 × FLAG plasmid was constructed by cloning the circBNC2 sequence into pcD-ciR (Geneseed Biotech) and a 3×FLAG-coding sequence was inserted at upstream of the stop codon (TGA) presented in the potential ORF sequence. If the potential ORF sequence was translated, a FLAG-tagged protein could be produced. Three days after transfection of circBNC2–3 × FLAG plasmid, HK2 cells were harvested and proteins were detected by western blot analysis with an anti-FLAG antibody.

### Gene gain-of-function and loss-of-function analysis in vivo

#### Gain-of-function for circBNC2

The full length of circBNC2 (circBNC2-FL) was inserted into a pK5ssAAV-ciR vector (Geneseed Biotech). After ensuring the accuracy of the vector by sequencing, adeno-associated virus serotype 9 (AAV9) was packaged, purified, and titrated by Genechem Biotechnology (Shanghai, China). AAV9 (1 × 10^11^ copies) carrying either the circBNC2 or the control sequence was injected through the left renal vein of mice to deliver circBNC2 to the left kidney or injected through the tail vein to deliver circBNC2 to the bilateral kidney and liver. Briefly, under general anesthesia, the left kidney of the mouse was exposed. The left renal vein was clamped by microaneurysm clamp, and AAV9 particles diluted in 100 μl of saline were injected into the left renal vein using a 31-gauge needle. The clamp was removed 15 min after injection followed by suturing of the incision.

To investigate whether AAV9-carrying circBNC2 was expressed in the kidney, a CMV-FLAG-Nanoluc-circBNC2 vector was constructed and packaged by AAV9 (Genechem). AAV9-FLAG-Nanoluc-circBNC2 was administrated by injection through the left renal vein or tail vein of mice. Eleven weeks after AAV9 administration, mice were sacrificed and the organ-specific luminescent imaging in kidney, heart, liver, spleen and lung was quantified by an IVIS Lumina III Imaging System (Caliper Life Science, USA). The FLAG labeled exogenic circBNC2 was examined by immunofluorescence assay and FISH assay in kidney sections from mice treated with AAV9 through the left renal vein.

#### Loss-of-function for circBNC2

The siRNA targeting circBNC2 was inserted into a GV478 vector and AAV9 was packaged, purified and titrated by Genechem Biotechnology. AAV9 (1 × 10^11^ copies) carrying either the siRNA or the scramble sequence was injected through the left renal vein of mice to knock down circBNC2 in the kidney.

### Pathologic studies

#### Mouse tissue samples

Mouse tissue samples, collected at the indicated times, were fixed in 10% formalin and embedded in paraffin or directly embedded in Tissue OCT-Freeze Medium (SAKURA).

#### Human study participants and human tissue samples

Human renal samples were obtained from renal biopsy specimens from Nanfang Hospital (*n* = 12 patients with IRI-induced renal fibrosis and *n* = 4 patients with AA-induced renal fibrosis) (Supplementary Table 3). Tissue adjacent to clear cell renal cell carcinoma were collected as the normal controls (*n* = 10). Liver biopsies of adult HBV-induced liver fibrosis patients (*n* = 10) were collected from Nanfang Hospital, Southern Medical University. Normal kidney or liver tissues were obtained from 10 patients undergoing tumorectomy. The study was approved by the Ethics Community of Nanfang hospital. All of the study participants provided written informed consent at the time of biopsy.

#### Masson’s staining

The kidney and liver sections (2 μm) were stained with Masson’s trichrome (Jiancheng Bioengineering Institute) according to the manufacturer’s protocol and scored by an experienced pathologist who was blind to the grouping. The degree of tubulointerstitial fibrosis for each sample was scored according to the fibrotic area: 0, no evidence of tubulointerstitial fibrosis; 1, <25% involvement; 2, 25–50% involvement; and 3, >50% involvement. The score of each kidney sample was presented as the mean of at least 10 random high-power (400×) fields.

The degree of liver fibrosis for each liver sample was semi-quantified by an experienced pathologist with Image-Pro Plus software (Media Cybernetics Bethesda, MD) and presented as the average percentages of at least 10 random high-power fields.

#### Immunohistochemistry

For immunohistochemistry staining, sections (4 μm) were incubated with antibodies against p-H3 (Ser 10) (Cell Signalling Technology, cat. 53348, dilution 1:100), αSMA (Cell Signalling Technology, cat. 19245, dilution 1:300), collagen I (Boster, China, cat. BA0325, dilution 1:300), FN (Sigma-Aldrich, SAB4500974, dilution 1:400) and ctBNC2 (customized by Sinobiotechnology, dilution 1:200) using microwave-based antigen retravel technique^38^. After washing with PBS buffer, the sections were detected by the EnVision/HRP Kit (Dako). The immunostaining was examined using an Olympus BX51 microscope (Olympus). To quantify the expression levels of αSMA, collagen I and Fibronectin in kidney sections, proportions of positively stained area in sections from each kidney were graded as follows: 0, no positive stained area; 1, <25%; 2, 25–50%; and 3, >50% area staining positive. The staining score in each kidney was the sum of scores of the 10 random fields. To quantify the p-H3 positive cell number in kidney and liver sections, the percentage of p-H3 positive staining cells was evaluated by an experienced pathologist with Image-Pro Plus software and presented as the average percentage of at least 10 random high-power fields. To quantify the collagen I and αSMA expression levels in liver sections, the percentage of positive staining area was evaluated with Image-Pro Plus software and presented as the average percentage of at least 10 random high-power fields.

#### TUNEL assay

For terminal deoxynucleotidyl transferase dUTP nick end labeling (TUNEL) staining, a DeadEnd Fluorometric TUNEL System was applied according to manufacturer’s instructions (G3250, Promega). The degree of hepatocyte apoptosis for each sample was quantified by an experienced pathologist with Image-Pro Plus software and presented as the average percentage of at least 10 random high-power fields.

### Dual-luciferase reporter system

To evaluate the activity of the IRES in circBNC2, the wild-type and mutant IRES sequences of circBNC2 were synthesized and cloned into Luc2-IRES-Report (Geneseed Biotech). HK2 cells were transfected with plasmids containing wild-type or mutant IRES sequences. The luciferase activity of Luc relative to that of RLuc was measured with dual-luciferase reporter gene assay kit (TransGen Biotech).

### Data analysis of CLIP-sequencing data for DHX9

The data from cross-linking immunoprecipitation-high-throughput sequencing (CLIP-sequencing) for DHX9 was retrieved from published studies^23^. Alignments were used to present read coverage of CLIP-sequencing data over the introns of BNC2. The Graphical representation was generated with the IGV genome browser^39^.

### Cross-linking RNA immunoprecipitation (RIP) assay

To verify the interaction of DHX9 with BNC2 pre-mRNA, cell lysates of 24 h hypoxia-treated HK2 cells were sonicated and immunoprecipitated with anti-DHX9 (Abcam, cat. Ab70777, dilution 1:100) and Magna RIP RNA-Binding Protein Immunoprecipitation Kit (Merk Millipore) according to manufacturer’s instructions. Co-precipitated RNA fragments were detected by qRT-PCR.

### RNA fluorescence in situ hybridization (FISH)

#### RNA FISH in cultured cells

To perform RNA FISH in cultured cells, LNA-modified circBNC2 probe (Exiqon) targeting the splice junction of circBNC2 were synthesized according to the sequences listed in Supplementary Table 2. The digoxin-conjugated probes for U6 were commercially available (Exiqon). RNA FISH in cells was performed as previously described with minor modification^38^. Briefly, cells were washed with PBS, fixed in 4% formaldehyde (Sigma-Aldrich) and permeabilized in PBS containing 0.1% Triton X-100. The cells on coverslips were incubated with probes targeting U6 or circBNC2 separately. Hybridizations were performed in hybridization solution (probe dilution 1:1000) (Boster) at 52 °C in a moist chamber for 16 h. After thoroughly washing in 25% deionized formamide/2×SSC at 52 °C, 30 min in 2×SSC. The cells were incubated with fluorescein labeled Digoxin antibody (Roche, cat. 11207741910, dilution 1:100) for 12 h at 4 °C and counterstained with DAPI.

#### RNA ISH and FISH in tissue

Expression of circBNC2 in animal kidneys, human renal and liver biopsy samples was examined with in situ hybridization. Briefly, sections of paraffin-embedded kidney tissue or cells cultured on coverslips were digested with trypsin (ZSGB-BIO, Beijing, China) and fixed in 4% paraformaldehyde, then hybridized with the digoxin labeled LNA-modified circBNC2 probe (Exiqon) at 52 °C for 16 h. After washing, samples were incubated overnight at 4 °C with anti-digoxin monoclonal antibody (Abcam, cat. ab419, dilution 1:200) followed by incubation of Alkaline Phosphatase Streptavidin (ZB2422, ZSGB-BIO) and then were stained with nitro blue tetrazolium/5-bromo-4-chloro-3-indolylphosphate (C3206, Beyotime) for RNA ISH assay. The proportion of positively circBNC2 stained cells in sections from each kidney and liver was counted under a 40× objective. The data were presented as the average percentages of at least 10 random high-power fields.

To perform RNA FISH in tissues, fresh OCT embedded kidney tissues were fixed in 4% formaldehyde and permeabilized in PBS containing 0.5% Triton X-100. Tissues were incubated with hybridization solution containing circBNC2 probe (1:1000) in a moist chamber after washing at 52 °C as described above, sections were incubated with rhodamine labeled Digoxin antibody (Roche, cat. 11207750910, dilution 1:100) and counterstained with DAPI.

To demonstrate that the exogenous circBNC2 carried by AAV9 was expressed in kidney cells, the renal cryosections from mice treated with AAV9-FLAG-Nanoluc-circBNC2 were fixed with 4% paraformalin fixing solution for 15 min at room temperature. After blocking with 10% normal goat serum in PBS, slides were incubated with primary antibody against FLAG (Sigma-Aldrich, cat. F7425, dilution 1:100). After washing with TBS-T, slides were incubated with Alexa Fluor 488-conjugated goat anti-rabbit IgG (Cell Signalling Technology, cat. 4412, dilution 1:200). Nuclei were stained with DAPI. Images were taken by confocal microscopy (Leica).

### ELISA

The concentrations of TGF-β and CTGF in cell supernatant were measured by ELISA. Briefly, cells were maintained in medium without serum for 48 h. Then the supernatants of cells were collected and measured according to the manufacturer’s instructions (R&D Systems), the analyses were performed using a VICTOR Multilabel Plate Reader (PerkinElmer).

### Western blot analysis

Cultured cells or tissues were lysed in RIPA buffer (Beyotime) with protease inhibitor (Sigma-Aldrich) and quantified using BCA protein quantitation kit (Thermo Fisher Scientific). The protein samples were subject to western blots as previously reported^38^. The primary antibodies used are as follows: anti-β-actin (Proteintech, cat. 20536-1-AP, dilution: 1:2000), anti-BNC2 (Novus, cat. NBP1-69078, dilution 1:1000), anti-αSMA (Cell Signaling Technology, cat. 19245, dilution 1:1000), anti-collagen I (Boster, cat. BA0325, dilution 1:1000), anti-FLAG (Sigma-Aldrich, cat. F1804, dilution 1:1000), anti-Fibronectin (Sigma-Aldrich, cat. SAB4500974, dilution 1:1000), anti-DHX9 (Abcam, cat. ab70777 or cat. ab26271, dilution 1:1000), anti-ADAR1 (Abcam, cat. ab168809, dilution 1:1000; Novus, cat. NBP2-92918, dilution 1:1000), anti-CDK1 (Cell Signaling Technology, cat. 9116, dilution 1:1000), anti-cyclin B1 (Cell Signaling Technology, cat. 12231, dilution 1:1000), anti-cyclin D1 (Cell Signaling Technology, cat. 55506, dilution 1:1000), anti-Lamin A/C (Cell Signaling Technology, cat. 4777, dilution 1:1000), anti-GAPDH (Cell Signaling Technology, cat. 5174, dilution 1:1000). To detect the expression of the protein translated by circBNC2, a polyclonal antibody against the specific amino acids sequence in the ctBNC2 was obtained by inoculating rabbits and purifying with affinity chromatography (customized by Sinobiotechnology, dilution 1:500). The HRP-linked secondary antibodies were purchased from Proteintech (Cat. SA00001-1 or SA00001-2, dilution 1:2000). In some experiments, to avoid detecting the heavy chain of IgG in the samples prepared by immunoprecipitation assay, light-chain specific HRP-linked secondary antibodies (Abbkine, cat. A25012 or A25022, dilution 1:1000) were used. Western blot images were quantitatively analyzed using the Image J program. The uncropped and unprocessed scans of western blot are provided in the Source Data file.

### Protein immunoprecipitation

Immunoprecipitations were conducted as previously described^38^. Briefly, human TECs or hepatocytes were lysed in IP lysis buffer (Thermo Fisher Scientific), then the lysates were centrifuged and cleared by incubation with protein A/G beads (Thermo Fisher Scientific) for 2 h at 4 °C on a rotator. The precleared supernatant was immunoprecipitated by anti-ctBNC2 (customized by Sinobiotechnology, dilution 1:100), anti-BNC2 (Novus, cat. NBP1-69078, dilution 1:100), anti-CDK1 (Cell Signalling Technology, cat. 9116, dilution 1:100), anti-cyclin B1 (Cell Signalling Technology, cat. 12231, dilution 1:100), anti-DHX9 (Abcam, cat. ab70777, dilution 1:100) and anti-FLAG (Sigma-Aldrich, cat. F1804, dilution 1:100). Then the immune complexes were co-immunoprecipitated by protein A/G beads (Invitrogen) followed by western blots. To determine the amino acids sequence of ctBNC2 that bound to cyclin B1 and CDK1, deletion mutations were constructed according to the amino acids sequence were listed in Supplementary Table 1. Plasmids (pcDNA3.1 vector) expressing FLAG-tagged truncated variants of ctBNC2 were transfected into HK2 cells for 48 h, separately. Then protein lysates of HK2 cells expressing truncated variants of ctBNC2 were collected and immunoprecipitations were performed with anti-FLAG (Sigma-Aldrich, dilution 1:100). Normal mouse IgG (Sigma-Aldrich, Cat# 12–371, dilution 1:100) and normal rabbit IgG (Cell Signaling Technology, Cat# 2729, dilution 1:100) were used as control in immunoprecipitation assays.

### Mass spectrometry analysis and immunoprecipitation-mass spectra analysis

To determine the expression of ctBNC2, LC-MS analysis was used with the silver-stained gel bands (70–100 kDa) from the lysates of TECs overexpressing circBNC2. In this experiment, three biological replicated samples (samples from three independent experiments, *n* = 1 for each experiment) were analyzed.

To identify the proteins interacted with full-length BNC2 or ctBNC2, LC-MS was performed with the silver-stained gel bands (25–35 and 55–70 kDa) containing complexes immoprecipitated by anti-BNC2 or anti-ctBNC2. In this experiment, three biological replicated samples (samples from three independent experiments, *n* = 1 for each experiment) were analyzed.

The LC-MS experiments were performed by Bioprofile (Shanghai, China). The relevant parameters of the LC-MS are as follows. The dried peptides were resuspended in 0.1% (v/v) formic acid solution. The peptides were separated by Easynano1200 LC (Thermo, San Jose, CA), equipped with 150 mm in-house C18 analytical column. The peptide mixture was loaded for 10 min at 3 μl/min on the trap column in 0.1% FA, then eluted to the analytical column and separated under the following conditions at 300 nl/min, 90 min running with a liquid gradient from 2 to 100% of solvent B (80% acetonitrile/0.1% FA) (2–45% buffer B for 80 min, 45–100% buffer B for 2 min, hold in 100% buffer B for 8 min). Mass spectrometer was performed on a mass spectrometer (Q Exactive HFX, Thermo Electron Finnigan, San Jose, CA). The voltage was set 2.1 kV for ESI by positive mode. A full MS survey scan (MS1) was set as a resolution of 60,000 at 200 m/z over the m/z range of 300–1800, Automatic gain controls (AGC) target of 3E6, maximum ion injection time (IT) of 50 ms. The top 20 multiply charged parent ions were selected by data dependent MS/MS mode, fragmented by the higher-energy collision dissociation (HCD) with the normalized collision energy of 30 at the m/z scan range of 100–2000. For the MS2 detection, the resolution was set at 15,000, AGC target value was 2E5, and the maximum IT was 60 ms. Dynamic exclusion was 30 s.

For peptide identification, the acquired data were analyzed against Homo sapiens Uniprot FASTA database (downloaded on 20220117) using Proteome Discoverer (version 2.4.1.15). Protease cleavage sites of trypsin were the C terminus of lysine and arginine with maximum missed cleavages set of 2. Carbamidomethylation of Cys was set as a fixed modification. Oxidation of Met and acetylation of protein N terminal were set as variable modifications. The mass tolerance for precursor and fragment ions were 4.5 and 20 ppm, respectively. The minimum peptide length was 6. The false discovery rate for peptide, protein, and site identification was set at 1%. The minimum number of unique peptides for protein identification was 1.

### Renal function and liver function

The plasma creatinine levels of mice were measured using the QuantiChrom Creatinine Assay Kit (DICT-500; Bioassay Systems) according to the manufacturer’s instructions. Serum alanine transaminase (ALT) levels were measured according to the manufacturer’s instructions, using a commercial kit (Nanjing Jiancheng Bioengineering institute).

To test the liver function, the hydroxyproline level in liver tissue was detected with Hydroxyproline assay kit (Jiancheng Bioengineering Institute) according to the manufacturer’s instructions.

### Statistics and reproducibility

The information about statistical details and methods is indicated in the figure legends or methods. Flow cytometry data were analyzed using FlowJo software. The measurements of all statistical values were performed using SPSS 23.0 or GraphPad Prism 7.0. Continuous variables were expressed as means ± SD. Data were tested for normality using the Kolmogorov–Smirnov test. To compare normally distributed continuous variables, we conducted a two-sided *T*-test or one-way ANOVA with Bonferroni post hoc test. For comparing variables that were not normally distributed, we used the two-sided Mann–Whitney *U* test. *P* < 0.05 was considered statistically significant.

The representative figures shown in Figs. 1d, f, g, i, k, 2a, i, p, w, 3d, n, 4e–h, m–p, q–s, u–y, 5b, h, k, o, u, d, j, q, t, 6b, d, f, h, k, l, o, and 7a, e and Supplementary Figs. 1b, c, i, 2j, l, m, n, p, u, 3d, h, 4b, c, j, l, 5a, f, h, j, k, p, and 6b, d, f, i, k, n, p were repeated at least three independent experiments with similar results.

### Reporting summary

Further information on research design is available in the Nature Research Reporting Summary linked to this article.

## Supplementary information

Supplementary Information

Description of Additional Supplementary Files

Supplementary Data 1

Supplementary Data 2

Supplementary Data 3

Reporting Summary

## Data Availability

All data associated with this study can be found in the paper or the Supplementary materials. RNA-sequencing data that supported the findings of this study have been deposited in GEO GSE179620, GSE179506, and GSE179505. All mass spectrometry raw data have been deposited to ProteomeXchange Consortium (http://proteomecentral.proteomexchange.org) via the iProX partner repository^40^ with the dataset identifier PXD033284. These data can be accessed by https://www.iprox.cn/page/project.html?id=IPX0004331000. The data that support the findings of this study are available from the corresponding authors upon reasonable request. Source Data are provided with this paper.

## Source data

Source Data
