# Supplementary material for: Circular RNA circBNC2 inhibits epithelial cell G2-M arrest to prevent fibrotic maladaptive repair
Source: Nat Commun. 2022 Oct 31;13:6502. doi: 10.1038/s41467-022-34287-5 (PMC9622807; doi:10.1038/s41467-022-34287-5)
Supplement: Supplementary file 1 — Supplementary Information [file 41467_2022_34287_MOESM1_ESM.pdf]

Circular RNA circBNC2 inhibits epithelial cell G2-M arrest to prevent fibrotic maladaptive repair

Peng Wang[1], Zhitao Huang[1], Yili Peng[1], Hongwei Li[1], Tong Lin[1], Yingyu Zhao[1], Zheng Hu[1], Zhanmei Zhou[1], Weijie Zhou[1], Youhua Liu[1], Fan Fan Hou[1,2]*

[1]Division of Nephrology, Nanfang Hospital, Southern Medical University, State Key Laboratory of Organ Failure Research, National Clinical Research Center of Kidney Disease, Guangdong Provincial Key Laboratory of Renal Failure Research.

[2]Guangzhou Regenerative Medicine and Health Guangdong Laboratory, Guangzhou 510515, China

* To whom correspondence should be addressed.

Fan Fan Hou, Division of Nephrology, Nanfang Hospital, Southern Medical University, ffhouguangzhou@163.com

Supplementary data

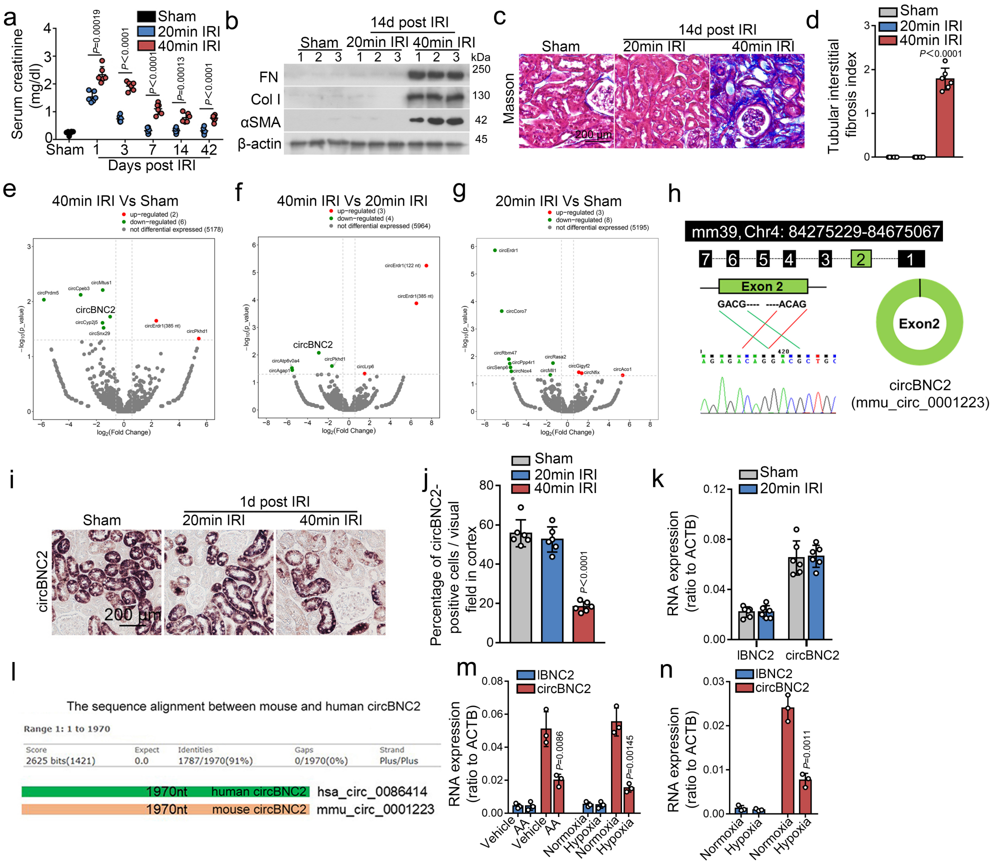

Supplementary Fig.1 Downregulation of circBNC2 is observed in severe IRI but not in mild IRI

a Changes of serum creatinine over time in IRI mouse (n=6 in each group). **b** Western blots showing fibronectin (FN), collagen I (Col I) and αSMA expression in kidney homogenates at day 14 post IRI. **c, d** Representative images (**c**) and quantification data (**d**) of Masson's trichrome staining in kidneys from mice subjected to mild (20-min ischemia) or severe (40-min ischemia) IRI at day 14 post injury. **e-g** Volcano plots showing the differentially expressed circRNAs in severe IRI V.s. Sham (e), severe IRI V.s. mild IRI (f) or mild IRI V.s. Sham (g). **h** The genomic localization and circBase ID for circBNC2. The backsplice junction site of circBNC2 was identified by Sanger sequencing. **i, j** Representative images (**i**) and quantification data (**j**) of *in situ* hybridization in kidney section from mice subjected to mild or severe IRI at day 1 post IRI. **k** RT-PCR showing circBNC2 and lBNC2 expression in mouse kidney at day 1 post mild IRI. **l** sequences of circBNC2 showing highly homologous (91%) between human and mouse. **m** qRT-PCR showing circBNC2 and lBNC2 expression in mouse TECs (mTECs) exposed to AA or hypoxia for 24 hours. **n** qRT-PCR showing circBNC2 and lBNC2 expression in 24-hour hypoxia-treated human hepatocytes (L-02). For **a, d**, **j**, **k**, n=6 mice in each group, for **m**, **n**, n=3 biologically independent cells. Data are expressed as means ± SD. Two-sided T-test was used for the comparison of two groups (**a**, **k**, **m**, **n**). One-way ANOVA with Bonferroni post hoc test was used for comparison among multiple groups (**d**, **j**). Source data are provided as a Source Data file.

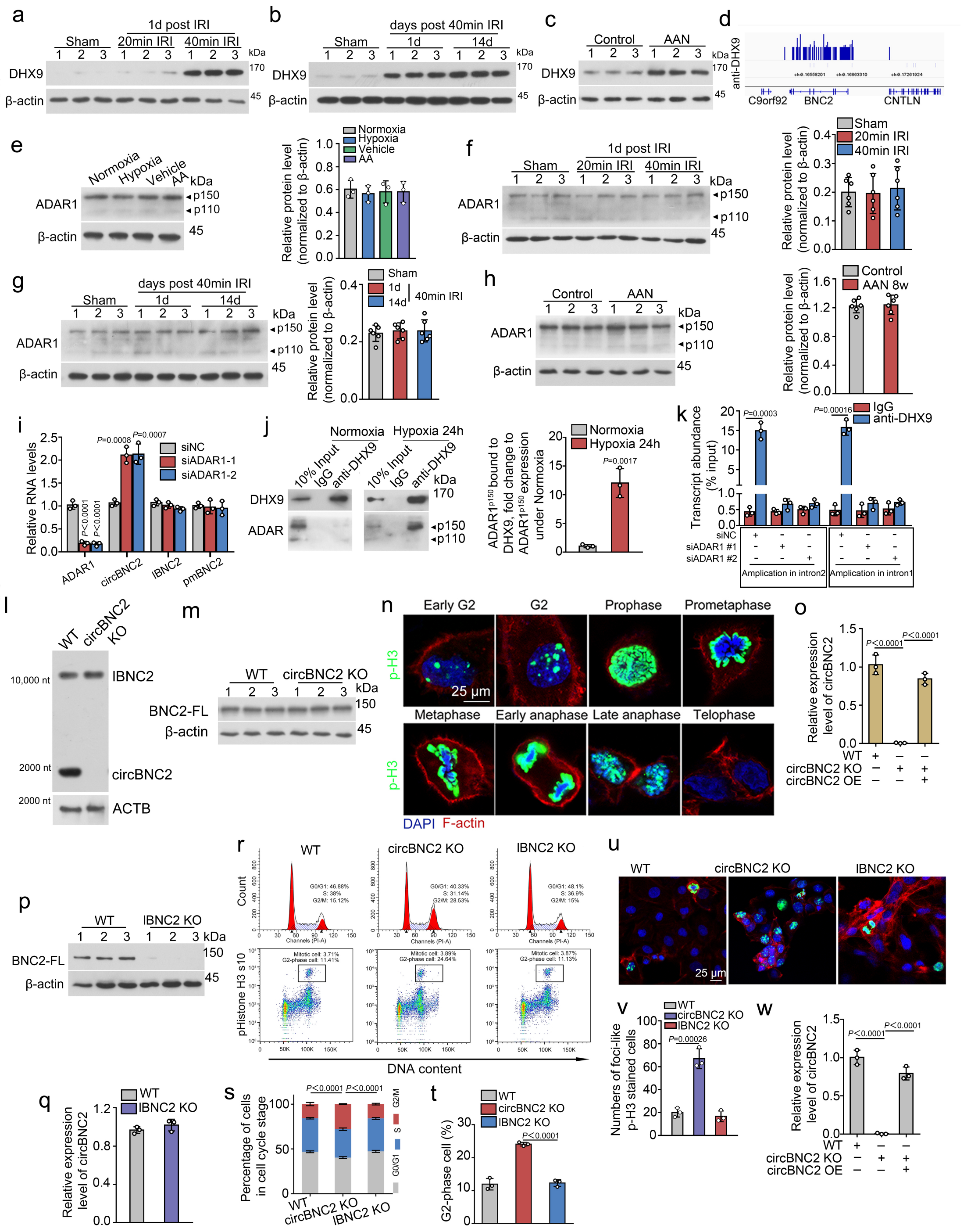

Supplementary Fig. 2 DHX9 interacts with ADAR1 to inhibit circBNC2 expression in injured TECs

a-c Western blots showing DHX9 expression in kidney from mice treated with severe IRI (**a, b**) and AA (**c**) (n=6 in each group). **d** Cross-linking-immunoprecipitation sequencing data showed the DHX9 binding sites within the introns of BNC2 pre-mRNA, according to the online CLIP-seq data. **e** Representative images of Western

blots and quantitative data showing the expression of ADAR1 in human TECs treated with hypoxia or aristolochic acids (AA) for 24 hours. **f** Representative images of Western blots and quantitative data showing the expression of ADAR1 in kidney homogenates from mice treated with mild or severe IRI at day 1 post injury (n=6 in each group). **g** Representative images of Western blots and quantitative data showing the expression of ADAR1 in kidney homogenates from mice treated with severe IRI at day 1 and day 14 post injury (n=6 in each group). **h** Representative images of Western blots and quantitative data showing the expression of ADAR1 in kidney homogenates from mice treated with AA at week 8 post injury (n=6 in each group). **i** qRT-PCR showing ADAR1, circBNC2, lBNC2 and pre-mRNA of BNC2 (pmBNC2) expression in 24 hours hypoxia-treated HK2 cells transfected with siRNAs targeting ADAR1. **j** Co-immunoprecipitation assays followed by Western blots showing that p150 isoform of ADAR1 bound to DHX9 in cell lysates from human TECs treated with hypoxia for 24 hours, and the quantification data. **k** RNA immunoprecipitation with anti-DHX9 in lysates from HK2 cells treated with hypoxia for 24 hours, followed by qRT-PCR assay. **l** Northern blot with probe targeting exon 2 of BNC2 showing expression of circBNC2 and lBNC2 in circBNC2-KO HK2 cells. **m** Representative images of Western blots showing the expression of full length BNC2 protein (BNC2-FL) in circBNC2-KO HK2 cells. **n** Chromatin patterns of TECs shown with immunostaining of antibody against p-Histone H3 *in vitro*. **o** qRT-PCR showing circBNC2 expression in circBNC2-KO HK2 cells transfected with circBNC2 plasmids. **p** Western blots showing the depletion of BNC2-FL protein in lBNC2-KO HK2. **q** qRT-PCR showing circBNC2 expression in lBNC2-KO HK2 cells. **r-t** Cell cycle analysis by flow cytometry in HK2 cells, showing knockout of circBNC2 induced G2/M cell cycle arrest (**r, s**), especially G2 phase cell (**t**), while depleting BNC2 protein had no effect on cell cycle regulation. **u, v** Immunofluorescence staining for p-H3 in circBNC2-KO HK2 cells showing increase in G2-phase positive cells (**u**) and the quantification data (**v**), while depleting BNC2 protein had no effect on G2-phase cell cycle arrest. **w** qRT-PCR showing circBNC2 expression in circBNC2-KO L-02 cells transfected with circBNC2 plasmids. For **e**, **i**, **j**,

o, **q**, **s**, **t**, **v**, **w**, n=3 biologically independent cells. For **f**, **g**, **h**, n=6 mice in each group. Data are expressed as means ± SD. Two-sided T-test was used for the comparison of two groups (**e**, **h**, **j**, **k**, **q**, **t**). One-way ANOVA with Bonferroni post hoc test was used for comparison among multiple groups (**f**, **g**, **i**, **o**, **s**, **v**, **w**). Source data are provided as a Source Data file.

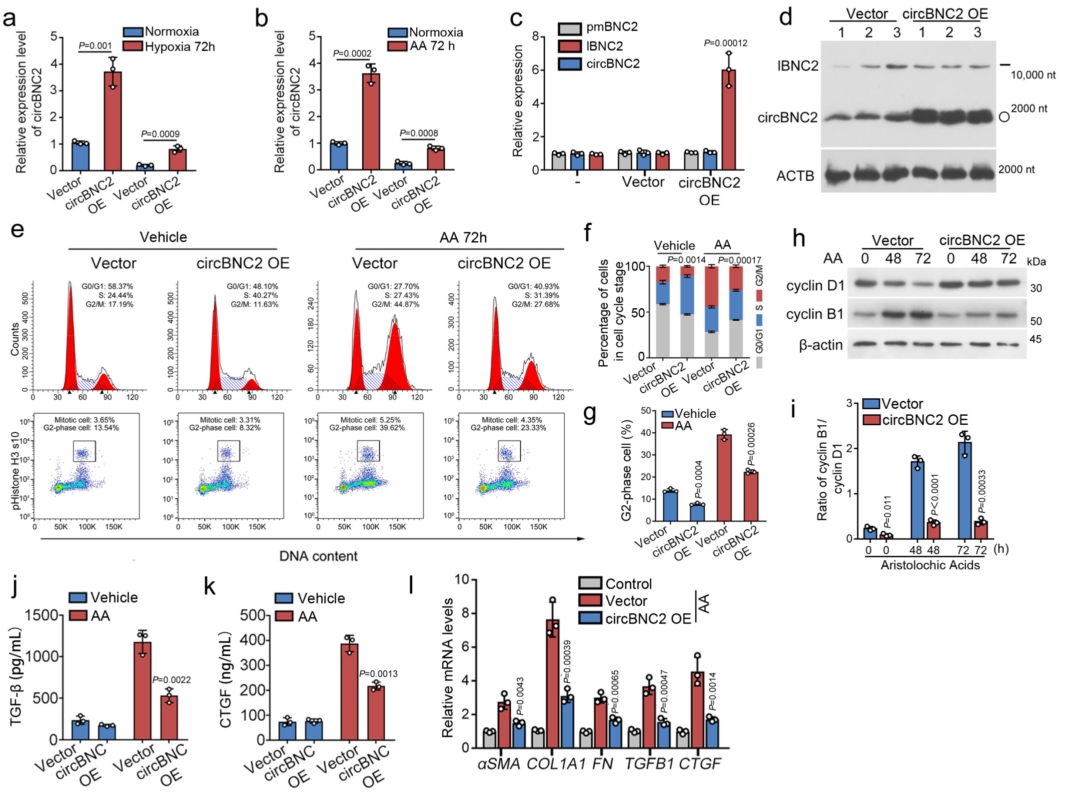

Supplementary Fig.3 Ectopic expression of circBNC2 attenuates epithelial cell G2/M arrest after exposure to aristolochic acids

a&b qRT-PCR showing efficacy of circBNC2 overexpression in HK2 cells treated with hypoxia (**a**) or AA (**b**) for 72 hours. **c** qRT-PCR showing expression levels of circBNC2, lBNC2 and pre-mRNA of BNC2 in HK2 cells transfected with circBNC2 overexpression lentivirus. **d** Northern blot with probe targeting exon 2 of BNC2 showing efficacy of circBNC2 transfection in HK2 cells. **e-g** Cell cycle analysis by flow cytometry in HK2 cells, showing overexpression of circBNC2 reduces G2/M cell cycle arrest induced by 72-hour AA treatment (**e, f**), especially G2 phase cell cycle

arrest (**g**). **h, i** Western blots showing the expression of cyclin B1 and cyclin D1 (**h**) and the quantification of cyclin B1/ cyclin D1 (**i**). **j, k** Levels of TGF-β1 (**j**) and CTGF (**k**) in culture medium, examined by ELISA, in AA-treated HK2 cells overexpressed with circBNC2. **l** qRT-PCR showing mRNA levels of profibrotic factors expression in AA-treated HK2 cells transfected with circBNC2 overexpression lentivirus. For **a-c**, **f**, **g**, **i-l**, n=3 biologically independent cells. Data are expressed as means ± SD. Two-sided T-test was used for the comparison of two groups (**a**, **b**, **f**, **g**, **i**, **j**, **k**). One-way ANOVA with Bonferroni post hoc test was used for comparison among multiple groups (**c**, **l**). Source data are provided as a Source Data file.

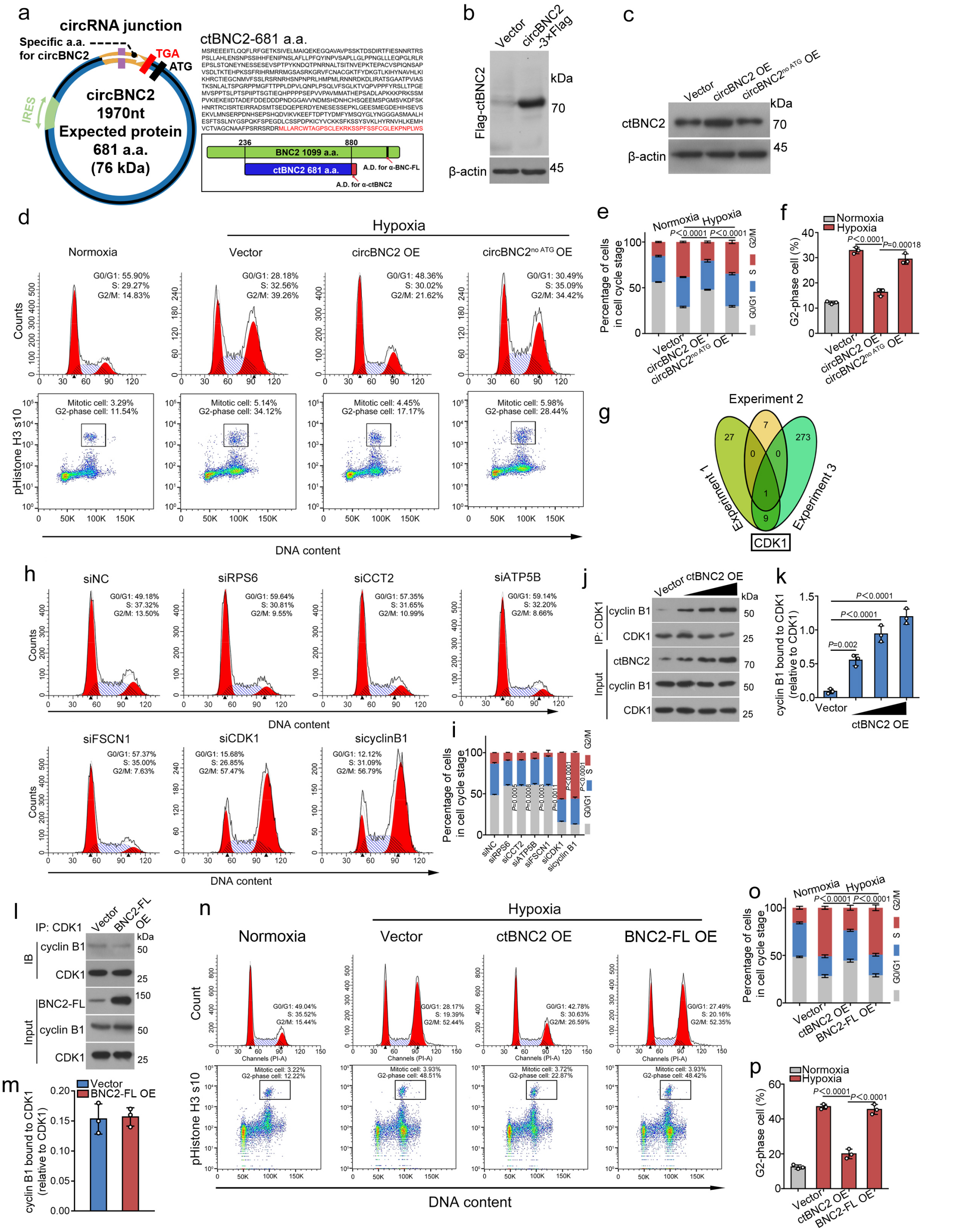

Supplementary Fig.4 circBNC2 encodes a 681-amino acid protein

a The amino acid sequence of the predicted 681-aa protein encoded by circBNC2 and the antigenic determinant (A.D.) for anti-ctBNC2 and anti-BNC2-FL. **b** Western blots showing FLAG-tagged-ctBNC2 in HK2 cells transfected with circBNC2-FLAG plasmid. **c** Western blots showing ctBNC2 in HK2 cells transfected with circBNC2 or circBNC2$^{no\ ATG}$ lentivirus. **d-f** Cell cycle analysis by flow cytometry in HK2 cells,

showing overexpression of circBNC2, but not circBNC2$^{no\ ATG}$, reduced 72-hour hypoxia-induced G2/M cell cycle arrest (**d, e**), especially G2 phase cell cycle arrest (**f**). **g** A Venn diagram showing the intersection presenting 1 protein (CDK1) bound to BNC2-FL in three replicated experiments. **h, i** Cell cycle analysis by flow cytometry in HK2 cells showed that knockdown of CDK1 or cyclin B1 induced G2/M cell cycle arrest, while knockdown of RPS6, CCT2, ATP5B or FSCN1 induced mild G0/G1 cell cycle arrest. **j, k** Interaction of CDK1 with cyclin B1 was increased by overexpressing ctBNC2 in 24-hour hypoxia-treated HK2 cells in a dose-dependent manner, as shown by Immunoprecipitation assay followed by Western blots. **l, m** Immunoprecipitation assay followed by Western blots showed that overexpressing BNC2-FL in 24-hour hypoxia-treated HK2 cells had no effect on the interaction of CDK1 with cyclin B1. **n-p** Cell cycle analysis by flow cytometry in HK2 cells, showing overexpression of ctBNC2, but not BNC2-FL, reduced 72-hour hypoxia-induced G2/M cell cycle arrest in HK2 cells (**n, o**), especially G2 phase cell cycle arrest (**p**). For **e**, **f**, **k**, **m**, **o**, **p**, n=3 biologically independent cells. Data are expressed as means ± SD. Two-sided T-test was used for the comparison of two groups (**i**, **m**). One-way ANOVA with Bonferroni post hoc test was used for comparison among multiple groups (**e**, **f**, **k**, **o**, **p**). Source data are provided as a Source Data file.

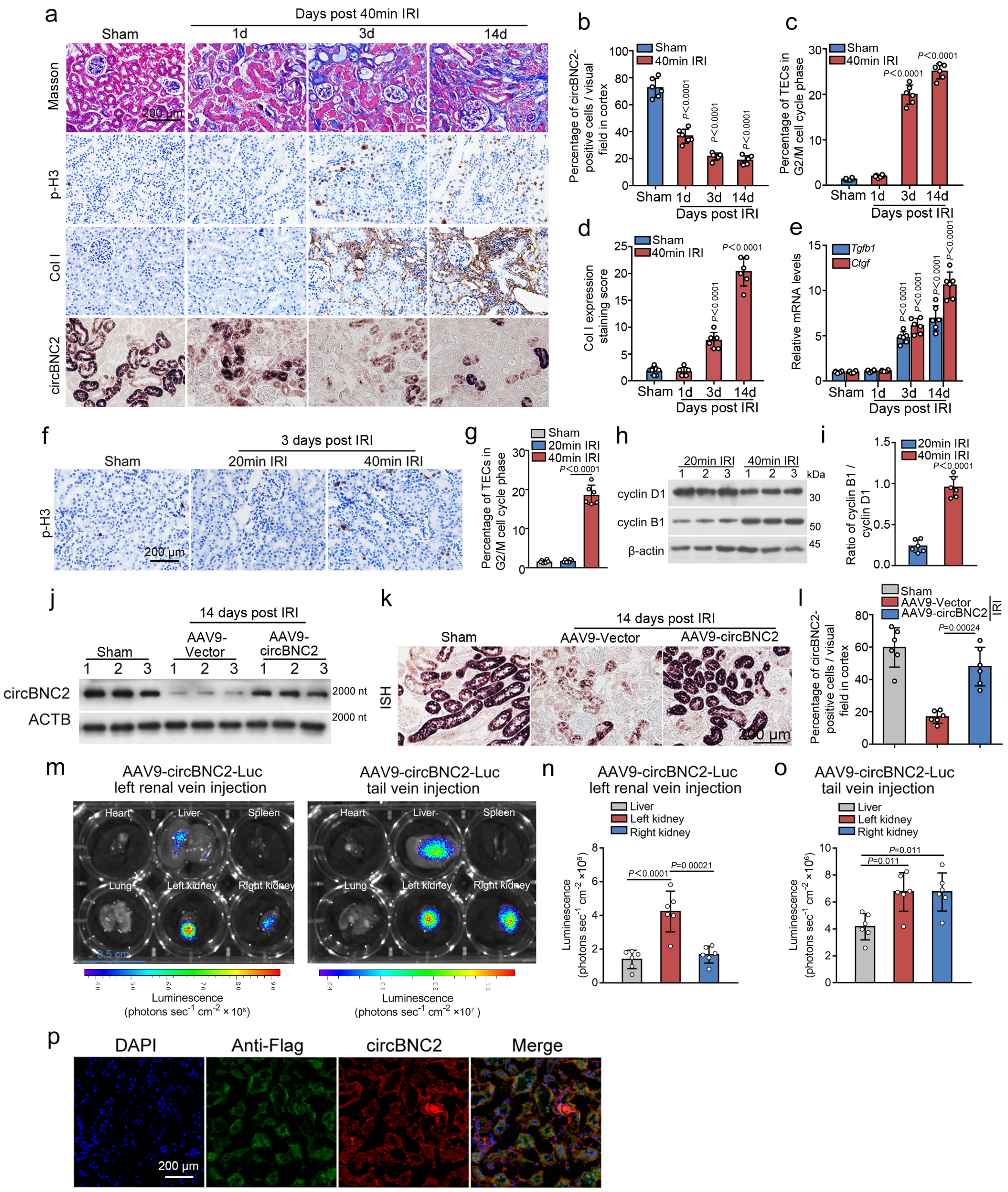

Supplementary Fig.5 circBNC2 alleviates fibrotic maladaptive repair in kidney after injury

a-d Representative images showing Masson staining, *in situ* hybridization *of* circBNC2, immunohistochemistry of p-H3 and Col I in kidney sections from mice treated with severe IRI (**a**) and the semi-quantitative data (**b-d**). **e** qRT-PCR assays showing mRNA levels of *Tgfb1* and *Ctgf* in kidney homogenates from mice treated with 40-min IRI. **f,**

g Representative photos showing expression of p-H3 in kidneys from mice treated with mild IRI or severe IRI (**f**) and the quantification data (**g**). **h, i** Western blots showing the expression of cyclin B1 and cyclin D1 (**h**) and the quantification of cyclin B1/ cyclin D1(**i**). **j** Northern blot with circBNC2 specific probe showing circBNC2 expression in IRI mice administrated with AAV9-circBNC2. **k, l** Representative images of *in situ* hybridization of circBNC2 expression in kidneys from IRI mice treated with AAV9-circBNC2 (**k**) and the percentage of circBNC2-positive TECs in renal cortex (**l**). **m-o** Representative images of luminescent imaging for kidney, heart, liver, lung and spleen from mice treated with AAV9-luciferase-circBNC2 through the left renal vein or tail vein (**m**) and quantification data for the photon intensities in the organs (**n, o**). **p** Representative images of Fluorescence *in situ* hybridization for circBNC2 followed by immunofluorescence for FLAG in renal sections from mice treated with AAV9-FLAG-circBNC2 through the left renal vein. For **b-e**, **g**, **i**, **l**, **n**, **o**, n=6 in each group. Data are expressed as means ± SD. Two-sided T-test was used for the comparison of two groups (**i**). One-way ANOVA with Bonferroni post hoc test was used for comparison among multiple groups (**b**, **c**, **d**, **e**, **g**, **l**, **n**, **o**). Source data are provided as a Source Data file.

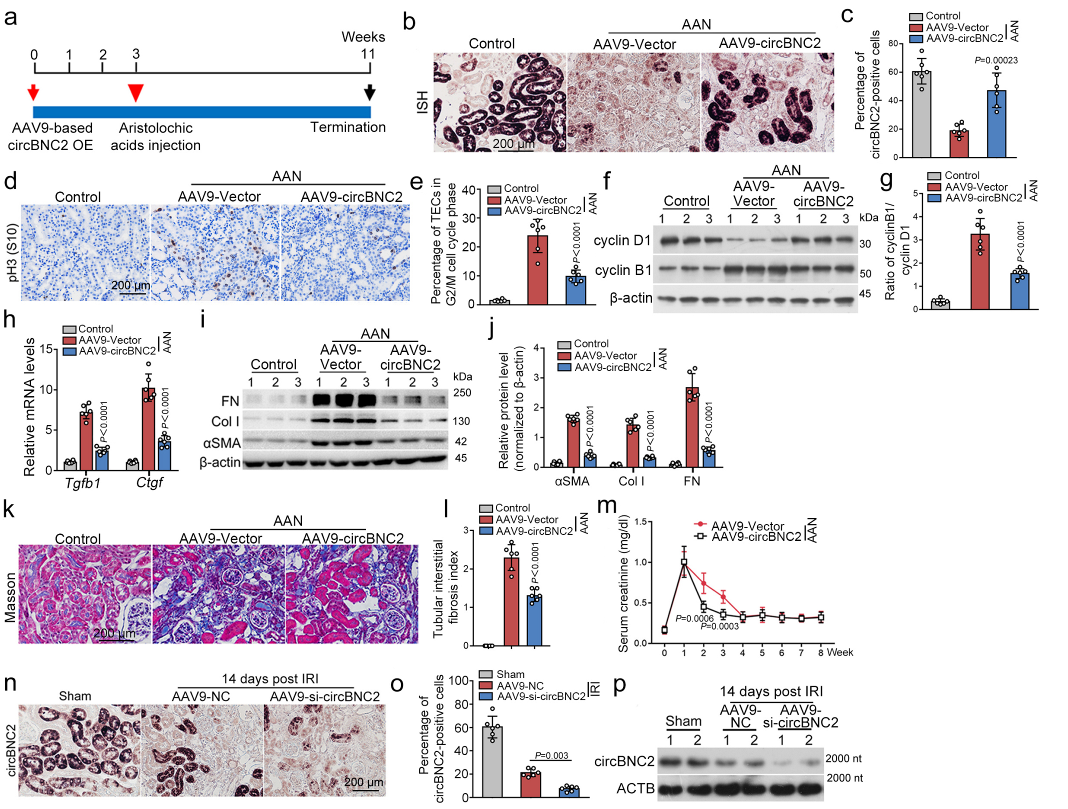

Supplementary Fig.6 Exogenous expression of circBNC2 alleviates fibrotic maladaptive repair in kidney after exposure to aristolochic acids

a Mice were treated by tail vein injection of either AAV9-circBNC2 or AAV9-Vector at 21 days before establishing AA nephropathy (AAN) model. Mice were euthanized 8 weeks post AAN model establishment (n=6 mice in each group). **b, c** Representative images of fluorescence *in situ* hybridization of circBNC2 expression in kidneys from AAN mice delivered with AAV9-circBNC2 (**b**) and the quantification data (**c**). **d, e** Representative images of Immunohistochemistry of p-H3 in AA-treated kidneys from AAN mice overexpressed with circBNC2 (**d**), and the quantification data (**e**). **f, g** Western blots showing expression of cyclin B1 and cyclin D1 in kidney homogenates from AAN mice overexpressed with circBNC2 (**f**), and the quantification data (**g**). **h** qRT-PCR showing the expression of *Tgfb1* and *Ctgf* in homogenates of kidney from AAN mice overexpressed with circBNC2. **i, j** Western blots showing expression of FN, collagen I and αSMA in kidneys from AAN mice treated with AAV9-circBNC2 (**i**), and the quantification data (**j**). **k, l** Masson staining of kidney sections from AAN mice

treated with AAV9-circBNC2 or AAV9-Vector (**k**), and the quantification data (**l**). **m** Administration of AAV-circBNC2 in AAN mice decreased the levels of serum creatinine at week 2 and 3, as compared to AAN mice treated with AAV9 vector. **n, o** Representative images of *in situ* hybridization of circBNC2 expression in kidneys from IRI mice delivered with AAV9-si-circBNC2 (**n**) and the quantification data (**o**). **p** Northern blot showing circBNC2 expression in IRI mice treated with AAV9-si-circBNC2 or AAV9-NC. For **c**, **e**, **g**, **h**, **j**, **l**, **m**, **o**, n=6 in each group. Data are expressed as means ± SD. Two-sided T-test was used for the comparison of two groups (**m**). One-way ANOVA with Bonferroni post hoc test was used for comparison among multiple groups (**c**, **e**, **g**, **h**, **j**, **l**, **o**). Source data are provided as a Source Data file.

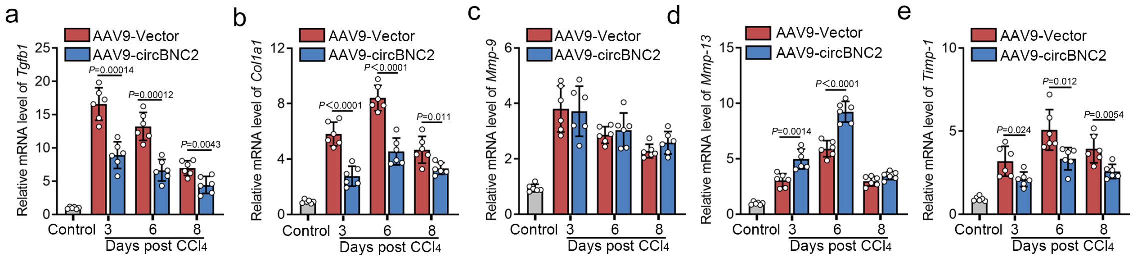

Supplementary Fig.7 Expression levels of fibrotic and fibrolytic genes in circBNC2 overexpressed mice following acute liver injury

a-e qRT-PCR assays showed that overexpressing circBNC2 reduced the mRNA levels of profibrotic factors, such as *Tgfb1*, *Col1a1* and *Timp-1*, and increased the mRNA level of *Mmp-13* at day 3, 6 and 8 post a single treatment of CCl_4 (n=6 in each group). Data are expressed as means ± SD. Two-sided T-test was used for the comparison of two groups. Source data are provided as a Source Data file.

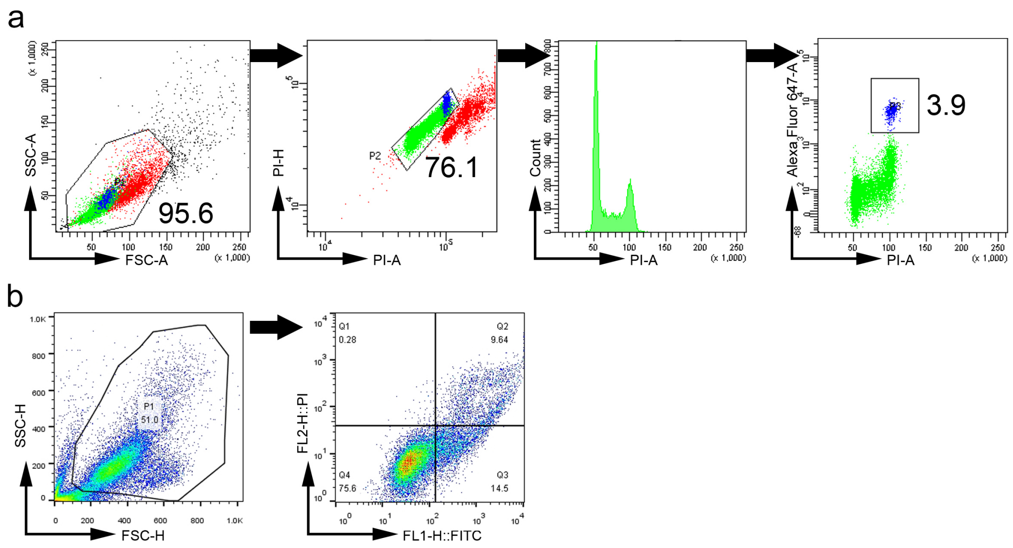

Supplementary Fig.8 Gating strategy for Flow Cytometry analysis

a Representative gating strategy for analyzing the G2/M cell cycle arrest. **b** Representative gating strategy for analyzing the apoptotic cells.

Supplementary Table 1. Sequences of truncated mutations of ctBNC2

Amino acids (aa) of ctBNC2	Sequence
**1-200 aa**	MSREEEIITLQQFLRFGETKSIVELMAIQEKEGQAVAVPSSKTDSDIRTFIESN NRTRSPSLLAHLENSNPSSIHHFENIPNSLAFLLPFQYINPVSAPLLGLPPNGL LLEQPGLRLREPSLSTQNEYNESSESEVSPTPYKNDQTPNRNALTSITNVEP KTEPACVSPIQNSAPVSDLTKTEHPKSSFRIHRMRRMGSA
**201-400 aa**	SRKGRVFCNACGKTFYDKGTLKIHYNAVHLKIKHRCTIEGCNMVFSSLRSR NRHSANPNPRLHMPMLRNNRDKDLIRATSGAATPVIASTKSNLALTSPGRP PMGFTTPPLDPVLQNPLPSQLVFSGLKTVQPVPPFYRSLLTPGEMVSPPTSL PTSPIIPTSGTIEQHPPPPSEPVVPAVMMATHEPSADLAPKKKPRK
**401-645 aa**	SSMPVKIEKEIIDTADEFDDEDDDPNDGGAVVNDMSHDNHCHSQEEMSPG MSVKDFSKHNRTRCISRTEIRRADSMTSEDQEPERDYENESESSEPKLGEES MEGDEHIHSEVSEKVLMNSERPDENHSEPSHQDVIKVKEEFTDPTYDMFY MSQYGLYNGGGASMAALHESFTSSLNYGSPQKFSPEGDLCSSPDPKICYVC KKSFKSSYSVKLHYRNVHLKEMHVCTVAGCNAAFPSRRSRDR
**401-481 aa**	SSMPVKIEKEIIDTADEFDDEDDDPNDGGAVVNDMSHDNHCHSQEEMSPG MSVKDFSKHNRTRCISRTEIRRADSMTSEDQEPERDYENESESSEPKLGEES MEGDEHIHSEVSEKVLMNSERPDENHSEPSHQDVIKVKEEFTDPTYDMFY MSQYGLYNGGGASMAALHESFTSSLNYGSPQKFSPEGDLCSSPDPKICYVC KKSFKSSYSVKLHYRNVHLKEMHVCTVAGCNAAFPSRRSRDRMLLARCW TAGPSCLEKRKSSPFSSFCGLEKPNPLWS
**1-645 aa**	MSREEEIITLQQFLRFGETKSIVELMAIQEKEGQAVAVPSSKTDSDIRTFIESN NRTRSPSLLAHLENSNPSSIHHFENIPNSLAFLLPFQYINPVSAPLLGLPPNGL LLEQPGLRLREPSLSTQNEYNESSESEVSPTPYKNDQTPNRNALTSITNVEP KTEPACVSPIQNSAPVSDLTKTEHPKSSFRIHRMRRMGSASRKGRVFCNAC GKTFYDKGTLKIHYNAVHLKIKHRCTIEGCNMVFSSLRSRNRHSANPNPRL HMPMLRNNRDKDLIRATSGAATPVIASTKSNLALTSPGRPPMGFTTPPLDP VLQNPLPSQLVFSGLKTVQPVPPFYRSLLTPGEMVSPPTSLPTSPIIPTSGTIE QHPPPPSEPVVPAVMMATHEPSADLAPKKKPRKSSMPVKIEKEIIDTADEFD DEDDDPNDGGAVVNDMSHDNHCHSQEEMSPGMSVKDFSKHNRTRCISRT EIRRADSMTSEDQEPERDYENESESSEPKLGEESMEGDEHIHSEVSEKVLM NSERPDENHSEPSHQDVIKVKEEFTDPTYDMFYMSQYGLYNGGGASMAA LHESFTSSLNYGSPQKFSPEGDLCSSPDPKICYVCKKSFKSSYSVKLHYRNV HLKEMHVCTVAGCNAAFPSRRSRDR
**1-681 aa**	MSREEEIITLQQFLRFGETKSIVELMAIQEKEGQAVAVPSSKTDSDIRTFIESN NRTRSPSLLAHLENSNPSSIHHFENIPNSLAFLLPFQYINPVSAPLLGLPPNGL LLEQPGLRLREPSLSTQNEYNESSESEVSPTPYKNDQTPNRNALTSITNVEP

KTEPACVSPIQNSAPVSDLTKTEHPKSSFRIHRMRRMGSASRKGRVFCNAC
GKTFYDKGTLKIHYNAVHLKIKHRCTIEGCNMVFSSLRSRNRHSANPNPRL
HMPMLRNNRDKDLIRATSGAATPVIASTKSNLALTSPGRPPMGFTTPPLDP
VLQNPLPSQLVFSGLKTVQPVPPFYRSLLTPGEMVSPPTSLPTSPIIPTSGTIE
QHPPPPSEPVVPAVMMATHEPSADLAPKKKPRKSSMPVKIEKEIIDTADEFD
DEDDDPNDGGAVVNDMSHDNHCHSQEEMSPGMSVKDFSKHNRTRCISRT
EIRRADSMTSEDQEPERDYENESESSEPKLGEESMEGDEHIHSEVSEKVLM
NSERPDENHSEPSHQDVIKVKEEFTDPTYDMFYMSQYGLYNGGGASMAA
LHESFTSSLNYGSPQKFSPEGDLCSSPDPKICYVCKKSFKSSYSVKLHYRNV
HLKEMHVCTVAGCNAAFPSRRSRDRMLLARCWTAGPSCLEKRKSSPFSSF
CGLEKPNPLWS

Supplementary Table 2. Primers, Probes and oligos used in this study.

Primer/ Probes/ oligos	Species	Sequence (5’ to 3’)	Amplified product (bp)
circBNC2	Human	Forward: AAAGAGATGCACGTCTGCAC<br>Reverse: GCAGAAACTGCTGAAGGGTG	147
lBNC2	Human	Forward: GCTCTGCCCACTTCTGTCAT<br>Reverse: GGCGCTTTCTCCCTCTCTTT	125
pmBNC2	Human	Forward: AGGAATGGAAAGCTTGAGGGTT<br>Reverse: AAAAAGCAAAGCCATGAGCCC	111
circBNC2	Mouse	Forward: AAAAGAGATGCACGTCTGCAC<br>Reverse: GCAGAAACTGCTGAAGGGTG	148
lBNC2	Mouse	Forward: CCCAGAACGGACACTCCAAA<br>Reverse: AGTGTAGACACAGAGGCACA	129
ACTB	Human	Forward: CATTCCAAATATGAGATGCGTTGT<br>Reverse: GCATTACATAATTTACACGAAAGC	148
ACTB	Mouse	Forward: TGAGCTGCGTTTTACACCCT<br>Reverse: GTTTGCTCCAACCAACTGCT	221
DHX9	Human	Forward: CGGTATGGAGATGGTCCACG<br>Reverse: CGCTACCATAGCCTCCACTG	130
ADAR1	Human	Forward: CACTTCCAGTGCGGAGTAGC<br>Reverse: GGTGTAGTATCCGCTGAGGG	157
TGF-β	Human	Forward: GCACCTTGGGCACTGTTGAA<br>Reverse: TCCCCCACTAAAGCAGGTTC	169
CTGF	Human	Forward: AGGATGTGCATTCTCCAGCC<br>Reverse: AAGTGGGGCTACCATTCTACT	135
αSMA	Human	Forward: GCTGCCCAGAGACCCTGTT<br>Reverse: TTTCATGGATGCCAGCAGACT	63
COL1A1	Human	Forward: GATCTGCGTCTGCGACAAC<br>Reverse: GGCAGTTCTTGGTCTCGTCA	68
SNAIL	Human	Forward: TCGGAAGCCTAACTACAGCGA	140

		Reverse: AGATGAGCATTGGCAGCGAG	
FN1	Mouse	Forward: TGTGATCCCCATGAAGCAACG<br>Reverse: CTCCGAAACACGTGCAGGA	109
TGF-β	Mouse	Forward: AGCTGCGCTTGCAGAGATTA<br>Reverse: TGCCGTACAACTCCAGTGAC	157
MMP-13	Mouse	Forward: ACCCAGCCCTATCCCTTGAT<br>Reverse: GGTCACGGGATGGATGTTCA	180
MMP-9	Mouse	Forward: GCGTCATTCGCGTGGATAAG<br>Reverse: CCTGGTTCACCTCATGGTCC	156
TIMP-1	Mouse	Forward: ACACCCCAGTCATGGAAAGC<br>Reverse: CTCAGAGTACGCCAGGGAAC	142
COL1A1	Mouse	Forward: CCCCTCAACCCCGTCTACTT<br>Reverse: ACAGTCCAAGAACCCCATGTC	70
αSMA	Mouse	Forward: CCCAGACATCAGGGAGTAATGG<br>Reverse: TCTATCGGATACTTCAGCGTCA	104
circPrdm5	Mouse	Forward: AAGAGGCTCGATCAAGTGGG<br>Reverse: TGGTCAGAGCTTGTTGCTCC	235
circCpeb3	Mouse	Forward: GCGCTTTGTGCAACTTCAAC<br>Reverse: ATCCTTGATGGTTGGGCTGG	153
circMtus1	Mouse	Forward: AGAACCCTAGGCTCCGACAG<br>Reverse: CGCGATCCATCTTCTGTCCT	265
circSnx29	Mouse	Forward: GTCCAGGCACTGGCCAGATGA<br>Reverse: GGCATGGCGCTCCTGCTGTT	187
circCyp2j5	Mouse	Forward: AATGCAAAAGCTCTACAATGGC<br>Reverse: GGGTTCCAATCTTTCTCGTGTTT	138
circPkhd1	Mouse	Forward: TCTTGATCTCTGGGTCAAACTG<br>Reverse: CAGGGTTCTGAGGGGAGACC	231
circErdr1	Mouse	Forward: AGATGTATGTGCCACCGACC<br>Reverse: TCCTACCTTGTGGAGTCCGT	191
primer pair c (intron 1) for DHX9 RIP assay	Human	Forward: AGGAATGGAAAGCTTGAGGGTT<br>Reverse: AAAAAGCAAAGCCATGAGCCC	111
primer pair b (intron 1) for DHX9 RIP assay	Human	Forward: TCTACACGGCCTGCCAAATC<br>Reverse: AGGAACAGCGAAGAAGCCAG	150
primer pair a (exon 3 and exon 4) for DHX9 RIP assay	Human	Forward: GTAAACATGGCTGGGTGGCA<br>Reverse: AGGATCTTTAGCCGCACAGG	157
primer pair d	Human	Forward: AAAGAGATGCACGTCTGCAC	147

for DHX9 RIP assay		Reverse: GCAGAAACTGCTGAAGGGTG	
DHX9 siRNA1	Human	GAGUGUAACAUCGUAGUAA	
DHX9 siRNA2	Human	CCCUGUCACUUGUCAGACA	
ADAR1 siRNA1	Human	GAAGACAGCAACUCCACAUCU	
ADAR1 siRNA2	Human	GUGCUUCAACACUCUGACUAA	
CDK1 siRNA	Human	GGAAUCUUUACAGGACUAUAA	
CCNB1 siRNA	Human	GUGACUGACAACACUUAUACU	
RPS6 siRNA	Human	GAGAGGGAAAGUUAGGUCAUA	
CCT2 siRNA	Human	GGACCCAAAGGCAUGGACAAA	
ATP5B siRNA	Human	GUGAGAGCACAGUAAGGACUA	
FSCN1 siRNA	Human	GAUCCAGUUCGGCCUCAUCAA	
circBNC2 siRNA	Mouse	GAAGCAGAGACAGGACGCU	
circBNC2 junction-site probe	Human	TTGCCAGCAGCATCCTGTCTCGGCTT-DIG	
circBNC2 Exon 2 probe	Human	TTAGATCACTGACTGGGGCAGAATTC-DIG	
circBNC2 probe	mouse	TTGCCAGCAGCGTCCTGTCTCTGCTT-DIG	
BNC2 probe	mouse	TTGCACATGATCCCACCATTGCTCCCA-DIG	
U6 probe	Human	CACGAATTTGCGTGTCATCCTT-DIG	
ACTB probe	Human	CTCATTGTAGAAGGTGTGGTGCCA-DIG	

Supplementary Table 3. Characteristics of patients with biopsy-proven IRI- or AAN-induced kidney fibrosis

Characteristics	Kidney fibrosis	
	IRI-induced (n=12)	Aristolochic acid-induced (n=4)
Male, n (%)	5 (41.7 %)	3 (75 %)
Female, n (%)	7 (58.3 %)	1 (25 %)
Age at biopsy, years	35.3 ± 7.8	60.3 ± 7.9
Systolic blood pressure, mm Hg	122.5 ± 8.0	118.8 ± 5.2
Diastolic blood pressure, mm Hg	72.9 ± 15.8	87.0 ± 9.7
Serum creatinine, μmol/L	147.6 ± 22.2	148.8 ± 12.1
eGFR, ml/min/1.73 m^2	45.5 ± 9.4	41.0 ± 7.5
Urinary protein excretion, g/day	1.8 ± 1.1	1.4 ± 0.6
