# Supplementary material for: Circular RNA circBNC2 inhibits epithelial cell G2-M arrest to prevent fibrotic maladaptive repair
Source: Nat Commun. 2022 Oct 31;13:6502. doi: 10.1038/s41467-022-34287-5 (PMC9622807; doi:10.1038/s41467-022-34287-5)
Supplement: Supplementary file 2 — Description of Additional Supplementary Files [file 41467_2022_34287_MOESM2_ESM.pdf]

Description of Additional Supplementary Files

File Name: Supplementary Data 1

Description: Mass spectrometry identifying proteins co-immunoprecipitated by anti-ctBNC2.

File Name: Supplementary Data 2

Description: Mass spectrometry identifying proteins co-immunoprecipitated by anti-BNC2-FL.

File Name: Supplementary Data 3

Description: MIQE checklist for qRT-PCR.
