# Supplementary material for: Circular RNA circBNC2 inhibits epithelial cell G2-M arrest to prevent fibrotic maladaptive repair
Source: Nat Commun. 2022 Oct 31;13:6502. doi: 10.1038/s41467-022-34287-5 (PMC9622807; doi:10.1038/s41467-022-34287-5)
Supplement: Supplementary file 6 — Reporting Summary [file 41467_2022_34287_MOESM6_ESM.pdf]

nature portfolio

Corresponding author(s): Fan Fan Hou

Last updated by author(s): Oct 11, 2022

Reporting Summary

Nature Portfolio wishes to improve the reproducibility of the work that we publish. This form provides structure for consistency and transparency in reporting. For further information on Nature Portfolio policies, see our Editorial Policies and the Editorial Policy Checklist.

Statistics

For all statistical analyses, confirm that the following items are present in the figure legend, table legend, main text, or Methods section.

n/a	Confirmed	
☐	☒	The exact sample size (*n*) for each experimental group/condition, given as a discrete number and unit of measurement
☐	☒	A statement on whether measurements were taken from distinct samples or whether the same sample was measured repeatedly
☐	☒	The statistical test(s) used AND whether they are one- or two-sided<br>*Only common tests should be described solely by name; describe more complex techniques in the Methods section.*
☒	☐	A description of all covariates tested
☐	☒	A description of any assumptions or corrections, such as tests of normality and adjustment for multiple comparisons
☐	☒	A full description of the statistical parameters including central tendency (e.g. means) or other basic estimates (e.g. regression coefficient) AND variation (e.g. standard deviation) or associated estimates of uncertainty (e.g. confidence intervals)
☒	☐	For null hypothesis testing, the test statistic (e.g. *F*, *t*, *r*) with confidence intervals, effect sizes, degrees of freedom and *P* value noted<br>*Give P values as exact values whenever suitable.*
☒	☐	For Bayesian analysis, information on the choice of priors and Markov chain Monte Carlo settings
☒	☐	For hierarchical and complex designs, identification of the appropriate level for tests and full reporting of outcomes
☒	☐	Estimates of effect sizes (e.g. Cohen's *d*, Pearson's *r*), indicating how they were calculated

Our web collection on statistics for biologists contains articles on many of the points above.

Software and code

Policy information about availability of computer code

Data collection	7500FAST Real-Time PCR System (Applied Biosystems) was used to collect quantitative PCR data. Mass spectrometer was performed on a mass spectrometer (Q Exactive HFX, Thermo Electron Finnigan, San Jose, CA). Luminescent imaging in kidney, heart, liver, spleen and lung was quantified by an IVIS Lumina III Imaging System (Caliper Life Science, USA). RNA-seq was performed by deep sequencing with an Illumina HiSeq 4000 (Aksomics, Shanghai).
Data analysis	GraphPad Prism 8 (GraphPad Software, USA) was used to draw graphs and analyze statistical data. BD FACSDIVA Software 4.1, FlowJo v10 (Tree Star, USA) and ModFit LT 5.0 (Verify Software House, USA) were used to analyze flow cytometry data. For peptide identification, the acquired data were analysed against Homo sapiens Uniprot FASTA database (downloaded on 20220117) using Proteome Discoverer (version 2.4.1.15). For the analysis of RNA-seq data, RSEM software package (1.3.1), FastQC (0.11.5), hisat2 (2.1.0), STAR software package (2.5.2b), CIRCexplorer2 (2.2.6) and edgeR (3.18.1) were used.

For manuscripts utilizing custom algorithms or software that are central to the research but not yet described in published literature, software must be made available to editors and reviewers. We strongly encourage code deposition in a community repository (e.g. GitHub). See the Nature Portfolio guidelines for submitting code & software for further information.

Data

Policy information about availability of data

All manuscripts must include a data availability statement. This statement should provide the following information, where applicable:

- Accession codes, unique identifiers, or web links for publicly available datasets
- A description of any restrictions on data availability
- For clinical datasets or third party data, please ensure that the statement adheres to our policy

RNA-sequencing data that supported the findings of this study have been deposited in GEO GSE179620 (https://www.ncbi.nlm.nih.gov/geo/query/acc.cgi?acc=GSE179620), GSE179506 (https://www.ncbi.nlm.nih.gov/geo/query/acc.cgi?acc=GSE179506) and GSE179505 (https://www.ncbi.nlm.nih.gov/geo/query/acc.cgi?acc=GSE179505). All mass spectrometry raw data have been deposited to ProteomeXchange Consortium (http://proteomecentral.proteomexchange.org) via the iProX partner repository with the dataset identifier PXD033284. These data can be accessed by this URL (https://www.iprox.cn/page/project.html?id=IPX0004331000). The data that support the findings of this study are available from the corresponding authors upon reasonable request.

Field-specific reporting

Please select the one below that is the best fit for your research. If you are not sure, read the appropriate sections before making your selection.

[x] Life sciences [ ] Behavioural & social sciences [ ] Ecological, evolutionary & environmental sciences

For a reference copy of the document with all sections, see nature.com/documents/nr-reporting-summary-flat.pdf

Life sciences study design

All studies must disclose on these points even when the disclosure is negative.

Sample size	Sample sizes for each experiment are described in figure legends. Sample sizes are determined empirically, and similar in size to most published studies in the same field (such as Nat Commun. 2022 Jan 21;13(1):438.). For in vitro assays, n=3. For animal experiments, usually n=5-8 mice were used.
Data exclusions	Generally no data were excluded, except in the animal experiments when 1-2 mice were dropped out for analyses due to accidental deaths of the mice.
Replication	Experiments were repeated with the same conditions and obtained similar results. The number of repeats were indicated in figure legends.
Randomization	Mice were randomly allocated among groups. For in vitro studies, the experiment was carried out strictly in accordance with the single variable principle. Treatment groups were divided randomly and equally.
Blinding	The investigators were not blinded for the in vitro experiments because the in vitro experiments were performed by several investigators, each of them was in charge for one specific experiment from sample preparation to data analysis, therefore, the investigators could not be blinded, because they had to know the treatment for each group. The fibrosis index of mice was scored by an experienced pathologist who was blinded to the grouping.

Reporting for specific materials, systems and methods

We require information from authors about some types of materials, experimental systems and methods used in many studies. Here, indicate whether each material, system or method listed is relevant to your study. If you are not sure if a list item applies to your research, read the appropriate section before selecting a response.

Materials & experimental systems

n/a	Involved in the study	
[ ]	[x]	Antibodies
[ ]	[x]	Eukaryotic cell lines
[x]	[ ]	Palaeontology and archaeology
[ ]	[x]	Animals and other organisms
[ ]	[x]	Human research participants
[ ]	[x]	Clinical data
[x]	[ ]	Dual use research of concern

Methods

n/a	Involved in the study	
[x]	[ ]	ChIP-seq
[ ]	[x]	Flow cytometry
[x]	[ ]	MRI-based neuroimaging

Antibodies

Antibodies used	1. Anti-human/mouse β-actin (Proteintech Cat# 20536-1-AP), dilution: 1:2000<br>2. Anti-human/mouse (BNC2 Novus Cat# NBP1-69078), dilution: 1:1000

3. Anti-human/mouse αSMA (Cell Signaling Technology Cat# 19245), dilution: 1:300
4. Anti-human/mouse collagen I (Boster Cat# BA0325), dilution: 1:300
5. Anti-FLAG (Sigma-Aldrich Cat# F1804), dilution: 1:1000 for Western blot; dilution: 1:100 for IP assay
6. Anti- human/mouse Fibronectin (Sigma-Aldrich Cat# SAB4500974), dilution: 1:400
7. Anti-human DHX9 (Abcam Cat# ab70777), dilution: 1:100 for RIP assay; dilution: 1:1000 for Western blot; dilution: 1:100 for IP assay
8. Anti-human/mouse DHX9 (Abcam Cat# ab26271), dilution: 1:1000
9. Anti-human ADAR1 (Abcam Cat# ab168809), dilution: 1:1000
10. Anti-mouse ADAR1 (Novus Cat# NBP2-92918), dilution: 1:1000
11. Anti- human/mouse CDK1 (Cell Signaling Technology Cat# 9116), dilution: 1:1000
12. Anti-human/mouse cyclin B1 (Cell Signaling Technology Cat# 12231), dilution: 1:1000
13. Anti-human/mouse cyclin D1 (Cell Signaling Technology Cat# 55506), dilution: 1:1000
14. Anti-human/mouse Phospho-Histone H3 (Ser10) (Cell Signaling Technology Cat# 53348), dilution: 1:100 for IHC; dilution: 1:200 for ISH; dilution: 1:800 for FCS;
15. Anti-human/mouse Lamin A/C (Cell Signaling Technology Cat# 4777), dilution: 1:2000
16. Anti-human/mouse GAPDH (Cell Signaling Technology Cat# 5174), dilution: 1:1000
17. Normal mouse IgG (Sigma-Aldrich Cat# 12-371), dilution: 1:100
18. Normal rabbit IgG (Cell Signaling Technology Cat# 2729), dilution: 1:100
19. HRP-linked goat anti-rabbit IgG antibody (Proteintech Cat# SA00001-2), dilution: 1:2000
20. HRP-linked goat anti-mouse IgG antibody (Proteintech Cat# SA00001-1), dilution: 1:2000
21. IPKine HRP AffiniPure Goat Anti-Mouse IgG Light Chain (Abbkine Cat# A25012), dilution: 1:1000
22. IPKine HRP AffiniPure Mouse Anti-Rabbit IgG Light Chain (Abbkine Cat# A25022), dilution: 1:1000
23. Goat anti-rabbit IgG H&L (Alexa Fluor 488) (Cell Signaling Technology Cat# 4412), dilution: 1:200 for ISH
24. Goat anti-rabbit IgG H&L (Alexa Fluor 647) (Cell Signaling Technology Cat# 4414), dilution: 1:500
25. Anti-digoxigenin-Rhodamine (Roche Cat# 11207750910), dilution: 1:100
26. Anti-digoxigenin-FITC (Roche Cat# 11207741910), dilution: 1:100
27. Biotin Anti-Digoxigenin antibody (Abcam Cat# ab419), dilution: 1:200

Validation

All antibodies in the study were freshly obtained from the manufacturers and used in accordance with the user manuals. All validation statements can be found on the respective antibody website.
1. β-actin: https://www.ptgcn.com/products/ACTB-Antibody-20536-1-AP.htm
2. BNC2: https://www.novusbio.com/products/bnc2-antibody_nbp1-69078
3. αSMA: https://www.cellsignal.cn/product/productDetail.jsp?productId=19245
4. Col I: https://www.boster.com.cn/home/product/anti-col1a1-antibody_ba0325.html
5. FLAG: https://www.sigmaaldrich.cn/CN/zh/product/sigma/f1804
6. FN: https://www.sigmaaldrich.cn/CN/zh/product/sigma/sab4500974
7. DHX9(H): https://www.abcam.cn/RNA-Helicase-A-antibody-ab70777.html
8. DHX9(H/M): https://www.abcam.cn/rna-helicase-a-antibody-ab26271.html
9. ADAR1(H): https://www.abcam.cn/adar1-antibody-ab168809.html
10. ADAR1(M): https://www.novusbio.com/products/adar-antibody_nbp2-92918
11. CDK1(H/M): https://www.cellsignal.cn/products/primary-antibodies/cdc2-poh1-mouse-mab/9116
12. cyclin B1: https://www.cellsignal.cn/products/primary-antibodies/cyclin-b1-d5c10-xp-rabbit-mab/12231
13. cyclin D1: https://www.cellsignal.cn/products/primary-antibodies/cyclin-d1-e3p5s-xp-rabbit-mab/55506
14. p-H3 (Ser10): https://www.cellsignal.cn/products/primary-antibodies/phospho-histone-h3-ser10-d7n8e-xp-rabbit-mab/53348?_=1664505882450&Ntt=53348&tahead=true
15. Lamin A/C: https://www.cellsignal.cn/products/primary-antibodies/lamin-a-c-4c11-mouse-mab/4777
16. GAPDH: https://www.cellsignal.cn/products/primary-antibodies/gapdh-d16h11-xp-rabbit-mab/5174
17. Normal mouse IgG: https://www.sigmaaldrich.cn/CN/zh/product/mm/12371
18. Normal rabbit IgG: https://www.cellsignal.cn/products/primary-antibodies/normal-rabbit-igg/2729?_=1664506725308&Ntt=2729&tahead=true
19. HRP-linked goat anti-rabbit IgG antibody: https://www.ptgcn.com/products/HRP-conjugated-Affinipure-Goat-Anti-Rabbit-IgG-H-L-secondary-antibody.htm#product-information
20. HRP-linked goat anti-mouse IgG antibody: https://www.ptgcn.com/products/HRP-conjugated-Affinipure-Goat-Anti-Mouse-IgG-H-L-secondary-antibody.htm
21. IPKine HRP AffiniPure Goat Anti-Mouse IgG Light Chain: https://www.abbkine.cn/product/a25012/
22. IPKine HRP AffiniPure Mouse Anti-Rabbit IgG Light Chain: https://www.abbkine.cn/product/a25022/
23. Goat anti-rabbit IgG H&L (Alexa Fluor 488): https://www.cellsignal.cn/products/secondary-antibodies/anti-rabbit-igg-h-l-f-ab-2-fragment-alexa-fluor-488-conjugate/4412
24. Goat anti-rabbit IgG H&L (Alexa Fluor 647) https://www.cellsignal.cn/products/secondary-antibodies/anti-rabbit-igg-h-l-f-ab-2-fragment-alexa-fluor-647-conjugate/4414?_=1664507043953&Ntt=4414&tahead=true
25. Anti-digoxigenin-Rhodamine: https://www.sigmaaldrich.cn/CN/zh/product/roche/11207750910
26. Anti-digoxigenin-FITC: https://www.sigmaaldrich.cn/CN/zh/product/roche/11207741910
27. Biotin Anti-Digoxigenin antibody: https://www.abcam.cn/biotin-digoxigenin-antibody-bt21h8-ab419.html

Eukaryotic cell lines

Policy information about cell lines

Cell line source(s)	Mouse immortalized tubule epithelial cell (mTEC) was a gift from H.-Y. Lan (The Chinese University of Hong Kong). HKC8 cells were a gift from L. Racusen (Johns Hopkins University). The conditionally immortalized mouse podocyte cell line (MPC-5) was established by Peter Mundel (Massachusetts General Hospital, Boston, MA) (Exp Cell Res. 1997 Oct 10;236(1):248-58).<br><br>The following cell lines used in this study were commercially available: the human tubular epithelial cell line HK2 [American Type Culture Collection (ATCC)], the human hepatocyte cell line L-02 (Shanghai Cell Bank, Shanghai, China), human embryonic kidney (HEK)-293T cells (Shanghai Cell Bank), human mesangial cells (ScienCell) and the human hepatic stellate cell line LX-2 (Merck Millipore). Mouse mesangial cells (sv40mes13) (Cellcook, Guangzhou, China).
Authentication	All cell lines were obtained from a trusted source and used at low passage. These cell lines were authenticated by the supplier using STR analysis.
Mycoplasma contamination	All cells were regularly tested for mycoplasma infection and were negative.
Commonly misidentified lines (See ICLAC register)	No commonly misidentified lines were used.

Animals and other organisms

Policy information about studies involving animals; ARRIVE guidelines recommended for reporting animal research

Laboratory animals	Male C57BL/6, aged 6 to 8 weeks (20-24 g) were used. Mice were housed in a standard environment which was characterized by 12 h light/dark cycle, 22-25 °C and 40-60% humidity with free access to water and forage. Animal welfare was reviewed during the application of the animal experiments by the Nanfang Hospital Animal Care Committee, and was monitored during the experiments by the employees of the Laboratory Animal Center of Nanfang Hospital. To euthanatize the mice before collecting tissue and blood samples, the mice were placed in a plexiglass chamber with 5% isoflurane (RWD, Cat #R510-22, Shenzhen) for 5 min. After that, cervical dislocation was performed when mice were fully sedated, as measured by a lack of active paw reflex.
Wild animals	No wild animals were used in the study.
Field-collected samples	No field-collected animals were used in the study.
Ethics oversight	All animal studies were approved by the Nanfang Hospital Animal Care Committee.

Note that full information on the approval of the study protocol must also be provided in the manuscript.

Human research participants

Policy information about studies involving human research participants

Population characteristics	Human renal samples were obtained from renal biopsy specimens from Nanfang Hospital (n=12 patients with IRI-induced renal fibrosis and n=4 patients with AA-induced renal fibrosis). Tissue adjacent to clear cell renal cell carcinoma were collected as the normal controls (n=10). Liver biopsies of adult HBV-induced liver fibrosis patients (n=10) were collected from Nanfang Hospital, Southern Medical University. Normal kidney or liver tissues were obtained from 10 patients undergoing tumorectomy. Tissue adjacent to tumor was collected as the normal controls. The study was approved by the Ethics Community of Nanfang hospital. All of the study participants provided written informed consent at the time of biopsy.<br><br>The 12 patients with IRI-induced renal fibrosis includes 5 male and 7 female patients in the 29-to-52 age range at the time of renal biopsy. The 4 patients with AA-induced renal fibrosis includes 3 male and 1 female patients in the 52-to-68 age range at the time of renal biopsy. The 10 patients with HBV-induced liver fibrosis includes 6 male and 4 female patients in the 38-to-53 age range at the time of liver biopsy.
Recruitment	The human tissue are obtained from biopsy samples. All the patients were provided with written informed consent and agreed that their tissue samples could be used for medical research.
Ethics oversight	The study was approved by the Ethics Community of Nanfang hospital.

Note that full information on the approval of the study protocol must also be provided in the manuscript.

Clinical data

Policy information about clinical studies

All manuscripts should comply with the ICMJEguidelines for publication of clinical research and a completedCONSORT checklist must be included with all submissions.

Clinical trial registration	n/a

Study protocol	n/a
Data collection	n/a
Outcomes	n/a

Flow Cytometry

Plots

Confirm that:

- [x] The axis labels state the marker and fluorochrome used (e.g. CD4-FITC).
- [x] The axis scales are clearly visible. Include numbers along axes only for bottom left plot of group (a 'group' is an analysis of identical markers).
- [x] All plots are contour plots with outliers or pseudocolor plots.
- [x] A numerical value for number of cells or percentage (with statistics) is provided.

Methodology

Sample preparation	For cell cycle assay, HK2 cells, L-02 and mouse TECs were trypsinized, centrifuged and fixed with 70% ice cold ethanol and stored at -20 ℃. 24 hours later, the fixed cells were stained with propidium iodine (PI) staining solution (Lianke). To quantify the percentage of M phase cells, the fixed cells were permeabilized with 0.3% TritonX-100 and incubated with anti-pH3 primary antibody (Cell Signaling) for 30 minutes and then stained with secondary antibody conjugated with Alexa Fluor 647 for 30 minute before the PI staining.<br><br>For apoptosis assay, cells were stained with annexin V and PI for 30 minutes with the FITC Annexin V Apoptosis Detection Kit II (BD Biosciences) according to the manufacturer's instructions.
Instrument	BD FACSCantoII
Software	BD FACSDIVATM Software 4.1
Cell population abundance	Cell population abundance is determined by FlowJo v10 (Tree Star, USA) and ModFit LT 5.0 (Verify Software House, USA)
Gating strategy	The FACS assay used in this study is an established protocol for cell cycle analysis and detection of apoptotic cells

- [x] Tick this box to confirm that a figure exemplifying the gating strategy is provided in the Supplementary Information.
